# The utility of texture analysis of kidney MRI for evaluating renal dysfunction with multiclass classification model

**DOI:** 10.1038/s41598-022-19009-7

**Published:** 2022-08-30

**Authors:** Yuki Hara, Keita Nagawa, Yuya Yamamoto, Kaiji Inoue, Kazuto Funakoshi, Tsutomu Inoue, Hirokazu Okada, Masahiro Ishikawa, Naoki Kobayashi, Eito Kozawa

**Affiliations:** 1 Department of Radiology, Saitama Medical University, 38 Morohongou, Moroyama-machi, Iruma-gun, Saitama Japan; 2 Department of Radiology, Tokyo Metropolitan Cancer and Infectious Diseases Center Komagome Hospital, 3-18-22 Honkomagome, Bunkyo-ku, Tokyo Japan; 3 Department of Nephrology, Saitama Medical University, 38 Morohongou, Moroyama-machi, Iruma-gun, Saitama Japan; 4 School of Biomedical Engineering, Faculty of Health and Medical Care, Saitama Medical University, 38 Morohongou, Moroyama-machi, Iruma-gun, Saitama Japan

**Keywords:** Nephrology, Kidney, Kidney diseases, Medical imaging, Magnetic resonance imaging

## Abstract

We evaluated a multiclass classification model to predict estimated glomerular filtration rate (eGFR) groups in chronic kidney disease (CKD) patients using magnetic resonance imaging (MRI) texture analysis (TA). We identified 166 CKD patients who underwent MRI comprising Dixon-based T1-weighted in-phase (IP)/opposed-phase (OP)/water-only (WO) images, apparent diffusion coefficient (ADC) maps, and T2* maps. The patients were divided into severe, moderate, and control groups based on eGFR borderlines of 30 and 60 mL/min/1.73 m^2^. After extracting 93 texture features (TFs), dimension reduction was performed using inter-observer reproducibility analysis and sequential feature selection (SFS) algorithm. Models were created using linear discriminant analysis (LDA); support vector machine (SVM) with linear, rbf, and sigmoid kernels; decision tree (DT); and random forest (RF) classifiers, with synthetic minority oversampling technique (SMOTE). Models underwent 100-time repeat nested cross-validation. Overall performances of our classification models were modest, and TA based on T1-weighted IP/OP/WO images provided better performance than those based on ADC and T2* maps. The most favorable result was observed in the T1-weighted WO image using RF classifier and the combination model was derived from all T1-weighted images using SVM classifier with rbf kernel. Among the selected TFs, total energy and energy had weak correlations with eGFR.

## Introduction

Chronic kidney disease (CKD) affects 8–16% of the population worldwide and remains a major threat to global public health due to its increasing incidence and mortality. Common causes of CKD include diabetes and hypertension, especially in developed countries. However, less than 5% of patients with early CKD report being aware of their disease^1^. Therefore, appropriate screening, early diagnosis, and management are significant in preventing CKD-associated adverse clinical outcomes, such as end-stage kidney disease, cardiovascular disease, and increased mortality. The Kidney Disease Improving Global Outcomes guidelines^2^ suggested a risk-based approach to the evaluation and management of CKD and proposed six disease categories related to the estimated glomerular filtration rate (eGFR): G1–G5, with G3 subdivided into 3a and 3b. The most essential cutoff points of eGFR are 60 and 30 mL/min/1.73 m^2^ (as the borderlines of G2/G3 and G3/G4, respectively); therefore, the risk of death was reported to increase as the eGFR decreased below 60 mL/min/1.73 m^2^ in recent CKD cohort studies^3^. In addition, an eGFR of less than 30 mL/min/1.73 m^2^ is important from a radiological point of view as it relates to the availability of the contrast media^4^. The risk stratification of CKD based on the eGFR has undisputed advantages and has helped achieve greater awareness of CKD and its impact on global health.

Ischemia and hypoxia are associated with the progression of CKD; however, clinical tools to quantify these factors in patients are lacking. Renal biopsy is the gold standard method to histologically evaluate renal pathology; nevertheless, it carries certain risks due to complications, such as bleeding. Conversely, magnetic resonance imaging (MRI) of the kidney has been used to non-invasively assess CKD progression. Several MRI methods have been successfully used to evaluate renal function, including diffusion-weighted imaging (DWI) and blood oxygen level-dependent imaging (BOLD). DWI and apparent diffusion coefficient (ADC) values are the most studied methods and have demonstrated a good correlation with renal function decline and renal fibrosis in CKD^5–8^. BOLD based on the T2* map reflects the regional renal oxygenation status and can assess hypoxia occurring during renal dysfunction^9,10^. Although Dixon-based gradient-echo MRI is another imaging method that is routinely performed in abdominal imaging and can measure renal lipid accumulation in type II diabetes mellitus^11^, its utility in the evaluation of CKD has not been thoroughly studied.

Texture analysis (TA) is an emerging technique that permits the quantification of image characteristics based on the distribution of pixels and their surface intensity or patterns^12,13^. TA has been applied to several medical image analyses, including oncologic imaging^14^, neuroimaging^15^, and abdominal imaging^16,17^. Recent reports have demonstrated the utility of TA based on DWI, BOLD, Susceptibility-weighted imaging (SWI), and T1 and T2 mapping^18,19^. However, TA of other essential MRI methods, including Dixon-based T1-weighted imaging (T1WI), has not been fully studied. Previous studies have described that as the renal function declines, a decreased difference between the values in the cortex and those in the medulla is observed in T1WI^20,21^. Therefore, we hypothesized that TA based on Dixon-based T1WI is especially important because of its capacity to capture the clearest images and reflect the morphological characteristics of the kidney.

Thus, this study aimed to assess and compare the performance of TA based on Dixon-based T1WI, ADC maps, and T2* maps (BOLD) for evaluating renal dysfunction.

## Results

### Clinical characteristics

The study included 166 participants. The major etiologies of CKD were hypertensive nephrosclerosis (n = 80), diabetic mellitus nephropathy (n = 25), immunoglobulin A (IgA) nephropathy (n = 22), and nephrotic syndrome (n = 5). No abnormalities were observed in the remaining 34 patients.

According to the eGFR, 36 patients had severe renal dysfunction (se-RD) (men, n [%] = 26 [72], mean age 60.9 ± 16.4 years, mean eGFR 19.8 ± 7.7 mL/min/1.73 m^2^), 85 patients had moderate renal dysfunction (mo-RD) (men, n [%] = 57 [67], mean age 62.3 ± 13.3 years, mean eGFR 46.3 ± 8.1 mL/min/1.73 m^2^), and 45 patients were in the control group (CG) (men, n [%] = 19 [42], mean age 43.7 ± 18.1 years, mean eGFR 78.1 ± 16.7 mL/min/1.73 m^2^).

Table 1 details the distribution of the study population in each eGFR group. The age, percentage of men, and incidence rates of hypertension and diabetes increased significantly with progressive renal dysfunction. There was no significant difference in the incidence rate of IgA nephropathy and nephrotic syndrome among the three groups.

**Table 1:** The demographic and clinical characteristics of the study population.

Variable	se-RD	mo-RD	CG	*P*
*N*	36	85	45	
Age, years, mean ± SD	60.9 ± 16.4	62.3 ± 13.3	43.7 ± 18.1	< 0.001
Sex, men, *n* (%)	26 (72)	57 (67)	19 (42)	0.006
Hypertension, *n* (%)	29 (81)	41 (48)	10 (22)	< 0.001
Diabetes, *n* (%)	12 (33)	11 (13)	2 (4)	< 0.001
IgA nephropathy, *n* (%)	5 (14)	10 (12)	7 (16)	0.54
Nephrotic syndrome, *n* (%)	1 (2.8)	2 (2)	2 (4.4)	0.51
eGFR, mL/min/1.73 m^2^, mean ± SD	19.8 ± 7.7	46.3 ± 8.1	78.1 ± 16.7	< 0.001

Unless otherwise indicated, data are represented as the number (%) of patients. *se-RD* severe renal dysfunction (estimated glomerular filtration rate [eGFR] < 30 mL/min/1.73 m^2^, i.e., CKD stage G4–5), *mo-RD* moderate renal dysfunction (30 ≤ eGFR < 60 mL/min/1.73 m^2^, i.e., CKD stage G3a/3b), *CG* control group (eGFR ≥ 60 mL/min/1.73 m^2^, i.e., CKD stage G1–2), *IgA* immunoglobulin A, *SD* standard deviation.

### Dimension reduction of texture features

The T1-weighted in-phase (IP)/opposed-phase (OP)/water-only (WO) images showed good reproducibility in the inter-observer reproducibility analysis, with mean interclass correlation coefficient (ICC) values of 0.767, 0.774, and 0.781, respectively. Conversely, the ADC and T2* maps showed slightly lower reproducibility, with mean ICC values of 0.732 and 0.718, respectively. Good inter-observer reproducibility was observed for 59, 60, 61, 54, and 50 features (ICC ≥ 0.75 and lower 95% confidence interval [CI] ≥ 0.6) in T1-weighted IP/OP/WO images, ADC map, and T2* map, respectively. By excluding features with poor reproducibility (ICC < 0.75 or lower 95% CI < 0.6) from any one of the imaging methods, the number of features for each imaging method was reduced to 40. Table 2 lists the ICC values of these 40 features for each imaging method.

**Table 2:** Representative texture features and their respective intraclass correlation coefficient.

Code	Feature class	Feature name code	Imaging method				
			T1WI IP	T1WI OP	T1WI WO	ADC map	T2* map
TF1	First-order	10th percentile	0.998	0.983	0.965	0.982	0.757
TF2	First-order	90th percentile	0.993	0.989	0.997	0.979	0.992
TF3	First-order	Energy	0.949	0.942	0.870	0.882	0.759
TF4	First-order	Entropy	0.905	0.912	0.912	0.887	0.919
TF5	First-order	Interquartile range	0.984	0.973	0.987	0.957	0.966
TF6	First-order	Mean absolute deviation	0.940	0.946	0.952	0.893	0.950
TF7	First-order	Mean	0.998	0.992	0.993	0.985	0.973
TF8	First-order	Median	0.999	0.995	0.996	0.987	0.979
TF9	First-order	Robust mean absolute deviation	0.982	0.975	0.986	0.951	0.971
TF10	First-order	Root mean squared	0.997	0.993	0.995	0.985	0.944
TF11	First-order	Total energy	0.987	0.943	0.872	0.948	0.800
TF12	First-order	Uniformity	0.951	0.949	0.961	0.930	0.849
TF13	GLCM	Difference average	0.857	0.883	0.805	0.872	0.917
TF14	GLCM	Difference entropy	0.849	0.874	0.808	0.846	0.948
TF15	GLCM	Id	0.948	0.953	0.936	0.937	0.956
TF16	GLCM	Idm	0.953	0.959	0.949	0.941	0.956
TF17	GLCM	Inverse variance	0.927	0.957	0.945	0.913	0.944
TF18	GLCM	Joint energy	0.940	0.946	0.955	0.937	0.856
TF19	GLCM	Joint entropy	0.887	0.910	0.890	0.905	0.935
TF20	GLCM	Maximum probability	0.955	0.957	0.972	0.949	0.855
TF21	GLCM	Sum entropy	0.938	0.931	0.931	0.903	0.908
TF22	GLDM	Dependence non uniformity	0.906	0.905	0.803	0.850	0.841
TF23	GLDM	Dependence non uniformity normalized	0.984	0.974	0.968	0.981	0.981
TF24	GLDM	Dependence variance	0.995	0.976	0.992	0.981	0.925
TF25	GLDM	Gray level non uniformity	0.974	0.965	0.987	0.985	0.846
TF26	GLDM	Large dependence emphasis	0.986	0.972	0.978	0.968	0.930
TF27	GLDM	Small dependence emphasis	0.956	0.964	0.951	0.940	0.957
TF28	GLRLM	Gray level non uniformity	0.963	0.967	0.983	0.983	0.772
TF29	GLRLM	Gray level non uniformity normalized	0.940	0.945	0.955	0.909	0.854
TF30	GLRLM	Long run emphasis	0.986	0.970	0.976	0.963	0.893
TF31	GLRLM	Run entropy	0.926	0.886	0.896	0.771	0.764
TF32	GLRLM	Run length non uniformity	0.880	0.916	0.833	0.801	0.786
TF33	GLRLM	Run length non uniformity normalized	0.970	0.969	0.964	0.951	0.948
TF34	GLRLM	Run percentage	0.980	0.971	0.971	0.963	0.941
TF35	GLRLM	Run variance	0.991	0.970	0.982	0.970	0.875
TF36	GLRLM	Short run emphasis	0.971	0.969	0.965	0.947	0.936
TF37	GLSZM	Gray level non uniformity normalized	0.883	0.925	0.929	0.799	0.845
TF38	GLSZM	Size zone non uniformity normalized	0.912	0.951	0.917	0.856	0.890
TF39	GLSZM	Small area emphasis	0.910	0.949	0.916	0.838	0.842
TF40	GLSZM	Zone percentage	0.970	0.968	0.964	0.953	0.952

*ADC* apparent diffusion coefficient, *GLCM* gray-level co-occurrence matrix, *GLDM* gray-level dependence matrix, *GLRLM* gray-level run length matrix, *GLSZM* gray-level size zone matrix, *IP* in-phase, *OP* opposed-phase, *TF* texture feature, *WO* water-only.

Subsequently, the sequential feature selection (SFS) algorithm was used for feature selection. For each imaging method and machine learning (ML) classifier, a subset of five features that provided good classification accuracies was identified. The selected features for each classification attempt are listed in Tables 3, 4, 5, 6 and 7.

**Table 3:** Performance of each classification attempt in discriminating between the three groups in T1-weighted in-phase imaging (T1WI IP).

	Accuracy (%)	Sensitivity (%)	Specificity (%)	AUC
**LDA (selected TFs = TF1, TF3, TF4, TF8, and TF20)**				
Macro-average	71.1 ± 1.0	56.7 ± 1.8	78.4 ± 1.0	0.764 ± 0.004
se-RD	70.0 ± 1.1	54.8 ± 1.5	77.6 ± 1.1	0.763 ± 0.004
mo-RD	64.4 ± 1.0	49.3 ± 2.3	72.0 ± 0.9	0.676 ± 0.007
CG	79.0 ± 1.1	66.0 ± 1.5	85.5 ± 0.9	0.840 ± 0.005
**SVM with linear kernel (selected TFs = TF3, TF11, TF13, TF20, and TF24)**				
Macro-average	75.0 ± 1.2	62.5 ± 1.8	81.2 ± 1.1	0.804 ± 0.005
se-RD	78.2 ± 1.2	65.0 ± 1.8	84.7 ± 1.0	0.836 ± 0.008
mo-RD	67.2 ± 1.0	55.0 ± 2.1	73.3 ± 1.2	0.702 ± 0.005
CG	79.7 ± 1.2	67.5 ± 1.4	85.7 ± 1.1	0.861 ± 0.007
**SVM with rbf kernel (selected TFs = TF3, TF11, TF20, TF28, and TF32)**				
Macro-average	78.8 ± 1.4	68.2 ± 2.1	84.1 ± 1.3	0.826 ± 0.006
se-RD	79.5 ± 1.3	68.2 ± 2.2	85.2 ± 1.6	0.865 ± 0.008
mo-RD	72.1 ± 1.3	64.3 ± 2.6	76.0 ± 1.4	0.729 ± 0.009
CG	84.7 ± 1.6	72.0 ± 1.6	91.0 ± 0.8	0.871 ± 0.005
**SVM with sigmoid kernel (selected TFs = TF1, TF5, TF22, TF25, and TF31)**				
Macro-average	74.2 ± 1.2	62.1 ± 1.9	80.7 ± 1.1	0.766 ± 0.005
se-RD	75.3 ± 1.2	68.7 ± 1.4	78.7 ± 1.3	0.780 ± 0.006
mo-RD	68.6 ± 1.1	45.3 ± 2.2	80.2 ± 1.2	0.659 ± 0.005
CG	78.9 ± 1.2	70.2 ± 2.0	83.2 ± 0.7	0.844 ± 0.005
**DT (selected TFs = TF3, TF11, TF18, TF24, and TF28)**				
Macro-average	78.2 ± 2.3	67.3 ± 4.0	83.6 ± 2.2	0.805 ± 0.012
se-RD	80.0 ± 2.5	72.6 ± 3.6	83.7 ± 2.0	0.835 ± 0.025
mo-RD	71.6 ± 2.1	57.4 ± 4.5	78.6 ± 2.8	0.716 ± 0.022
CG	83.0 ± 2.5	71.8 ± 3.9	88.6 ± 1.9	0.863 ± 0.019
**RF (selected TFs = TF3, TF5, TF9, TF25, and TF28)**				
Macro-average	81.2 ± 1.6	71.7 ± 2.6	85.9 ± 1.5	0.871 ± 0.005
se-RD	82.8 ± 1.6	74.2 ± 2.9	87.2 ± 1.2	0.901 ± 0.005
mo-RD	76.9 ± 1.5	62.5 ± 2.8	84.1 ± 1.8	0.802 ± 0.009
CG	83.7 ± 1.7	78.4 ± 2.1	86.3 ± 1.4	0.898 ± 0.005

*TF* texture feature, *DT* decision tree, *LDA* linear discriminant analysis, *SVM* support vector machine, *RF* random forest classifier, *AUC* area under the curve, *se-RD* severe renal dysfunction (estimated glomerular filtration rate [eGFR] < 30 mL/min/1.73 m^2^, i.e., CKD stage G4–5), *mo-RD* moderate renal dysfunction (30 ≤ eGFR < 60 mL/min/1.73 m^2^, i.e., CKD stage G3a/3b), *CG* control group (eGFR ≥ 60 mL/min/1.73 m^2^, i.e., CKD stage G1–2). Feature name codes are as follows: TF1 = 10th percentile, TF3 = energy, TF4 = entropy, TF5 = interquartile range, TF8 = median, TF9 = robust mean absolute deviation, TF11 = total energy, TF13 = difference average, TF18 = joint energy, TF20 = maximum probability, TF22 = dependence non uniformity, TF24 = dependence variance, TF25 = gray level non uniformity (gray-level dependence matrix), TF28 = gray level non uniformity (gray-level run length matrix), TF31 = run entropy, TF32 = run length non uniformity. The data are expressed as means ± standard deviations.

**Table 4:** Performance of each classification attempt in discriminating between the three groups in T1-weighted opposed-phase imaging (T1WI OP).

	Accuracy (%)	Sensitivity (%)	Specificity (%)	AUC
**LDA (selected TFs = TF2, TF3, TF13, TF22, and TF24)**				
Macro-average	76.4 ± 0.7	64.5 ± 1.2	82.3 ± 0.8	0.813 ± 0.003
se-RD	75.9 ± 0.8	63.7 ± 0.9	82.0 ± 0.7	0.816 ± 0.005
mo-RD	70.1 ± 0.7	49.6 ± 1.8	80.4 ± 0.7	0.732 ± 0.006
CG	83.1 ± 0.7	80.2 ± 1.0	84.5 ± 0.9	0.879 ± 0.004
**SVM with linear kernel (selected TFs = TF3, TF5, TF15, TF22, and TF25)**				
Macro-average	76.2 ± 1.1	64.3 ± 2.1	82.1 ± 1.1	0.782 ± 0.005
se-RD	74.3 ± 1.1	63.2 ± 1.9	79.8 ± 1.3	0.777 ± 0.007
mo-RD	70.9 ± 0.9	49.0 ± 2.6	81.9 ± 1.3	0.691 ± 0.005
CG	83.4 ± 1.2	80.6 ± 1.7	84.7 ± 0.8	0.864 ± 0.008
**SVM with rbf kernel (selected TFs = TF2, TF3, TF11, TF16, and TF17)**				
Macro-average	77.2 ± 1.0	65.8 ± 1.7	82.9 ± 1.0	0.766 ± 0.005
se-RD	77.6 ± 1.0	64.6 ± 2.2	84.1 ± 0.9	0.775 ± 0.008
mo-RD	71.4 ± 0.9	60.8 ± 1.8	76.7 ± 1.4	0.670 ± 0.009
CG	82.6 ± 1.1	72.0 ± 1.1	87.9 ± 0,6	0.840 ± 0.007
**SVM with sigmoid kernel (selected TFs = TF2, TF3, TF24, TF25, and TF31)**				
Macro-average	76.2 ± 1.2	67.7 ± 2.0	83.9 ± 1.1	0.812 ± 0.006
se-RD	74.3 ± 1.1	71.2 ± 1.6	82.6 ± 1.1	0.813 ± 0.008
mo-RD	70.9 ± 0.9	54.6 ± 2.5	80.7 ± 1.4	0.720 ± 0.006
CG	83.4 ± 1.2	77.4 ± 1.8	88.3 ± 0.8	0.890 ± 0.008
**DT (selected TFs = TF3, TF13, TF24, TF26, and TF34)**				
Macro-average	76.6 ± 2.0	64.8 ± 4.1	82.4 ± 2.5	0.792 ± 0.012
se-RD	78.3 ± 2.1	59.9 ± 3.3	87.4 ± 2.7	0.804 ± 0.022
mo-RD	69.8 ± 1.8	63.4 ± 5.1	73.0 ± 2.9	0.723 ± 0.020
CG	81.6 ± 2.1	71.2 ± 3.8	86.8 ± 2.0	0.848 ± 0.018
**RF (selected TFs = TF3, TF7, TF10, TF17, and TF38)**				
Macro-average	81.0 ± 1.5	71.6 ± 2.2	85.8 ± 1.3	0.869 ± 0.005
se-RD	83.3 ± 1.5	74.7 ± 2.4	87.7 ± 1.4	0.894 ± 0.006
mo-RD	75.4 ± 1.3	67.5 ± 2.6	79.4 ± 1.4	0.805 ± 0.009
CG	84.4 ± 1.7	72.5 ± 1.7	90.3 ± 1.0	0.895 ± 0.004

*TF* texture feature, *DT* decision tree, *LDA* linear discriminant analysis, *SVM* support vector machine, *RF* random forest classifier, *AUC* area under the curve, *se-RD* severe renal dysfunction (estimated glomerular filtration rate [eGFR] < 30 mL/min/1.73 m^2^, i.e., CKD stage G4–5), *mo-RD* moderate renal dysfunction (30 ≤ eGFR < 60 mL/min/1.73 m^2^, i.e., CKD stage G3a/3b), *CG* control group (eGFR ≥ 60 mL/min/1.73 m^2^, i.e., CKD stage G1–2). Feature name codes are as follows: TF2 = 90th percentile, TF3 = energy, TF5 = interquartile range, TF7 = mean, TF10 = root mean squared, TF11 = total energy, TF13 = difference average, TF15 = id, TF16 = idm, TF17 = inverse variance, TF22 = dependence non uniformity, TF24 = dependence variance, TF25 = gray level non uniformity (gray-level dependence matrix), TF26 = large dependence emphasis, TF31 = run entropy, TF34 = run percentage, TF38 = size zone non uniformity normalized. The data are expressed as means ± standard deviations.

**Table 5:** Performance of each classification attempt in discriminating between the three groups in T1-weighted water-only imaging (T1WI WO).

	Accuracy (%)	Sensitivity (%)	Specificity (%)	AUC
**LDA (selected TFs = TF3, TF6, TF12, TF19, and TF24)**				
Macro-average	76.8 ± 0.8	65.3 ± 1.3	82.6 ± 0.8	0.824 ± 0.003
se-RD	81.4 ± 0.9	73.4 ± 1.4	85.4 ± 0.7	0.862 ± 0.004
mo-RD	71.1 ± 0.8	55.3 ± 1.4	79.1 ± 0.9	0.752 ± 0.006
CG	78.0 ± 0.9	67.1 ± 1.2	83.4 ± 0.7	0.844 ± 0.004
**SVM with linear kernel (selected TFs = TF5, TF11, TF18, TF24, and TF32)**				
Macro-average	76.7 ± 1.2	65.0 ± 2.1	82.5 ± 1.2	0.834 ± 0.005
se-RD	81.9 ± 1.2	76.5 ± 1.7	84.5 ± 0.9	0.887 ± 0.005
mo-RD	69.0 ± 1.1	51.9 ± 2.4	77.5 ± 1.4	0.741 ± 0.006
CG	79.1 ± 1.2	66.5 ± 2.2	85.4 ± 1.1	0.860 ± 0.006
**SVM with rbf kernel (selected TFs = TF3, TF13, TF18, TF32, and TF39)**				
Macro-average	78.9 ± 1.6	68.4 ± 2.8	84.2 ± 1.6	0.832 ± 0.005
se-RD	83.3 ± 1.7	71.7 ± 2.8	89.1 ± 1.6	0.881 ± 0.007
mo-RD	70.0 ± 1.4	57.4 ± 3.1	76.4 ± 1.8	0.712 ± 0.005
CG	83.3 ± 1.7	76.0 ± 2.6	87.0 ± 1.3	0.890 ± 0.005
**SVM with sigmoid kernel (selected TFs = TF1, TF13, TF22, TF31, and TF32)**				
Macro-average	76.5 ± 1.6	64.7 ± 2.6	82.3 ± 1.5	0.812 ± 0.006
se-RD	79.6 ± 1.7	70.1 ± 3.3	84.3 ± 1.1	0.844 ± 0.007
mo-RD	70.1 ± 1.5	54.0 ± 2.6	78.1 ± 1.9	0.724 ± 0.006
CG	79.7 ± 1.7	70.0 ± 1.8	84.6 ± 1.4	0.853 ± 0.007
**DT (selected TFs = TF3, TF12, TF24, TF29, and TF30)**				
Macro-average	81.3 ± 1.7	71.9 ± 3.0	86.0 ± 1.3	0.818 ± 0.012
se-RD	83.9 ± 1.7	67.3 ± 3.0	92.1 ± 2.0	0.853 ± 0.017
mo-RD	75.1 ± 1.5	74.0 ± 3.4	75.6 ± 1.8	0.743 ± 0.021
CG	85.0 ± 1.9	74.5 ± 2.6	90.2 ± 1.2	0.855 ± 0.020
**RF (selected TFs = TF11, TF14, TF16, TF29, and TF32)**				
Macro-average	82.0 ± 1.6	73.0 ± 2.6	86.5 ± 1.4	0.884 ± 0.005
se-RD	86.7 ± 1.7	78.4 ± 2.3	90.9 ± 1.3	0.924 ± 0.006
mo-RD	75.6 ± 1.4	63.0 ± 3.3	81.9 ± 1.6	0.809 ± 0.009
CG	83.6 ± 1.7	77.5 ± 2.5	86.7 ± 1.4	0.907 ± 0.005

*TF* texture feature, *DT* decision tree, *LDA* linear discriminant analysis, *SVM* support vector machine, *RF* random forest classifier, *AUC* area under the curve, *se-RD* severe renal dysfunction (estimated glomerular filtration rate [eGFR] < 30 mL/min/1.73 m^2^, i.e., CKD stage G4–5), *mo-RD* moderate renal dysfunction (30 ≤ eGFR < 60 mL/min/1.73 m^2^, i.e., CKD stage G3a/3b), *CG* control group (eGFR ≥ 60 mL/min/1.73 m^2^, i.e., CKD stage G1–2). Feature name codes are as follows: TF1 = 10th percentile, TF3 = energy, TF5 = interquartile range, TF6 = mean absolute deviation, TF11 = total energy, TF12 = uniformity, TF13 = difference average, TF14 = difference entropy, TF16 = idm, TF18 = joint energy, TF19 = joint entropy, TF22 = dependence non uniformity, TF24 = dependence variance, TF29 = gray level non uniformity normalized, TF30 = long run emphasis, TF31 = run entropy, TF32 = run length non uniformity, TF39 = small area emphasis. The data are expressed as means ± standard deviations.

**Table 6:** Performance of each classification attempt in discriminating between the three groups in ADC map imaging.

	Accuracy (%)	Sensitivity (%)	Specificity (%)	AUC
**LDA (selected TFs = TF3, TF13, TF15, TF27, and TF36)**				
Macro-average	70.6 ± 1.0	55.9 ± 1.7	77.9 ± 1.1	0.748 ± 0.004
se-RD	72.7 ± 1.0	63.7 ± 1.6	77.2 ± 1.1	0.785 ± 0.006
mo-RD	62.6 ± 0.9	34.9 ± 2.3	76.5 ± 1.2	0.619 ± 0.008
CG	76.4 ± 1.1	69.0 ± 1.3	80.1 ± 0.9	0.828 ± 0.004
**SVM with linear kernel (selected TFs = TF3, TF6, TF16, TF29, and TF37)**				
Macro-average	69.9 ± 1.3	54.8 ± 2.1	77.4 ± 1.3	0.736 ± 0.006
se-RD	69.0 ± 1.2	60.7 ± 2.4	73.2 ± 1.5	0.781 ± 0.007
mo-RD	61.2 ± 1.1	32.6 ± 2.4	75.5 ± 1.6	0.573 ± 0.006
CG	79.3 ± 1.4	71.1 ± 1.6	83.5 ± 0.7	0.842 ± 0.008
**SVM with rbf kernel (selected TFs = TF3, TF15, TF30, TF38, and TF39)**				
Macro-average	72.1 ± 1.6	58.1 ± 2.9	79.3 ± 1.9	0.757 ± 0.007
se-RD	74.6 ± 1.6	64.0 ± 2.9	80.0 ± 1.7	0.803 ± 0.009
mo-RD	65.0 ± 1.5	45.0 ± 3.4	75.0 ± 2.2	0.633 ± 0.008
CG	76.6 ± 1.7	65.3 ± 2.3	82.2 ± 1.7	0.823 ± 0.010
**SVM with sigmoid kernel (selected TFs = TF3, TF11, TF25, TF28, and TF39)**				
Macro-average	69.2 ± 1.6	53.8 ± 3.5	76.9 ± 2.3	0.696 ± 0.006
se-RD	67.8 ± 1.5	71.8 ± 4.8	65.8 ± 2.1	0.739 ± 0.006
mo-RD	63.5 ± 1.4	14.4 ± 3.8	88.0 ± 3.2	0.529 ± 0.006
CG	76.4 ± 1.8	75.3 ± 1.9	77.0 ± 1.5	0.808 ± 0.010
**DT (selected TFs = TF13, TF14, TF25, TF33, and TF36)**				
Macro-average	70.0 ± 2.5	55.0 ± 4.5	77.5 ± 2.7	0.713 ± 0.014
se-RD	74.2 ± 2.7	72.7 ± 3.9	75.0 ± 2.6	0.791 ± 0.020
mo-RD	62.2 ± 2.3	33.8 ± 4.6	76.4 ± 3.1	0.574 ± 0.026
CG	73.5 ± 2.6	58.4 ± 5.0	81.1 ± 2.3	0.773 ± 0.021
**RF (selected TFs = TF4, TF11, TF15, TF16, and TF25)**				
Macro-average	75.0 ± 1.5	62.4 ± 2.7	81.2 ± 1.5	0.808 ± 0.005
se-RD	75.4 ± 1.5	73.0 ± 2.7	76.6 ± 1.6	0.843 ± 0.008
mo-RD	68.8 ± 1.3	39.3 ± 3.4	83.5 ± 1.6	0.699 ± 0.010
CG	80.7 ± 1.6	75.0 ± 2.0	83.5 ± 1.3	0.870 ± 0.006

*TF* texture feature, *DT* decision tree, *LDA* linear discriminant analysis, *SVM* support vector machine, *RF* random forest classifier, *AUC* area under the curve, *se-RD* severe renal dysfunction (estimated glomerular filtration rate; eGFR < 30 mL/min/1.73 m^2^, i.e., CKD stage G4–5), *mo-RD* moderate renal dysfunction (30 ≤ eGFR < 60 mL/min/1.73 m^2^, i.e., CKD stage G3a/3b), *CG* control group (eGFR ≥ 60 mL/min/1.73 m^2^, i.e., CKD stage G1–2). Feature name codes are as follows: TF3 = energy, TF4 = entropy, TF6 = mean absolute deviation, TF11 = total energy, TF13 = difference average, TF14 = difference entropy, TF15 = id, TF16 = idm, TF25 = gray level non uniformity (gray-level dependence matrix), TF27 = small dependence emphasis, TF28 = gray level non uniformity (gray-level run length matrix), TF29 = gray level non uniformity normalized, TF30 = long run emphasis, TF33 = run length non uniformity normalized, TF36 = short run emphasis, TF37 = gray level non uniformity normalized, TF38 = size zone non uniformity normalized, TF39 = small area emphasis. The data are expressed as means ± standard deviations.

**Table 7:** Performance of each classification attempt in discriminating between the three groups in T2* map imaging.

	Accuracy (%)	Sensitivity (%)	Specificity (%)	AUC
**LDA (selected TFs = TF2, TF11, TF22, TF24, and TF38)**				
Macro-average	73.2 ± 0.7	59.9 ± 1.3	80.0 ± 1.1	0.737 ± 0.004
se-RD	71.4 ± 0.7	61.0 ± 1.6	76.7 ± 1.2	0.740 ± 0.006
mo-RD	71.0 ± 0.7	50.7 ± 1.5	81.2 ± 1.2	0.667 ± 0.007
CG	77.3 ± 0.8	67.9 ± 0.9	82.0 ± 0.8	0.792 ± 0.004
**SVM with linear kernel (selected TFs = TF2, TF3, TF10, TF11, and TF35)**				
Macro-average	67.1 ± 1.3	50.7 ± 2.4	75.4 ± 1.7	0.694 ± 0.006
se-RD	62.5 ± 1.2	63.5 ± 3.0	62.0 ± 2.0	0.689 ± 0.006
mo-RD	63.0 ± 1.2	23.4 ± 3.3	82.9 ± 2.0	0.578 ± 0.007
CG	75.9 ± 1.5	65.1 ± 1.0	81.2 ± 1.0	0.802 ± 0.010
**SVM with rbf kernel (selected TFs = TF3, TF6, TF11, TF13, and TF22)**				
Macro-average	71.9 ± 1.7	57.8 ± 3.2	78.9 ± 2.1	0.739 ± 0.007
se-RD	72.5 ± 1.6	56.0 ± 3.6	80.7 ± 2.4	0.751 ± 0.010
mo-RD	63.9 ± 1.6	54.8 ± 4.2	68.4 ± 2.0	0.642 ± 0.012
CG	79.2 ± 1.9	62.5 ± 1.7	87.6 ± 1.8	0.811 ± 0.007
**SVM with sigmoid kernel (selected TFs = TF3, TF5, TF7, TF11, and TF26)**				
Macro-average	69.8 ± 1.7	54.7 ± 3.0	77.4 ± 1.9	0.729 ± 0.007
se-RD	68.1 ± 1.7	57.5 ± 3.1	73.4 ± 2.4	0.747 ± 0.008
mo-RD	62.4 ± 1.5	39.3 ± 3.9	74.0 ± 1.9	0.590 ± 0.006
CG	78.9 ± 1.9	67.3 ± 2.0	84.7 ± 1.4	0.837 ± 0.009
**DT (selected TFs = TF3, TF13, TF25, TF32, and TF40)**				
Macro-average	69.8 ± 2.4	54.7 ± 4.9	77.3 ± 3.2	0.721 ± 0.014
se-RD	69.9 ± 2.4	53.0 ± 5.0	78.4 ± 3.5	0.743 ± 0.023
mo-RD	61.8 ± 2.2	46.0 ± 6.2	69.8 ± 3.4	0.620 ± 0.024
CG	77.6 ± 2.7	65.0 ± 3.4	83.8 ± 2.8	0.798 ± 0.018
**RF (selected TFs = TF6, TF10, TF11, TF13, and TF22)**				
Macro-average	74.9 ± 2.1	62.3 ± 3.5	81.1 ± 1.9	0.821 ± 0.006
se-RD	76.0 ± 2.0	63.6 ± 3.6	82.2 ± 1.9	0.832 ± 0.009
mo-RD	67.5 ± 1.8	51.2 ± 4.1	75.6 ± 2.3	0.725 ± 0.014
CG	81.1 ± 2.3	72.1 ± 2.8	85.6 ± 1.4	0.895 ± 0.006

*TF* texture feature, *DT* decision tree, *LDA* linear discriminant analysis, *SVM* support vector machine, *RF* random forest classifier, *AUC* area under the curve, *se-RD* severe renal dysfunction (estimated glomerular filtration rate [eGFR] < 30 mL/min/1.73 m^2^, i.e., CKD stage G4–5), *mo-RD* moderate renal dysfunction (30 ≤ eGFR < 60 mL/min/1.73 m^2^, i.e., CKD stage G3a/3b), *CG* control group (eGFR ≥ 60 mL/min/1.73 m^2^, i.e., CKD stage G1–2). Feature name codes are as follows: TF2 = 90th percentile, TF3 = energy, TF5 = interquartile range, TF6 = mean absolute deviation, TF7 = mean, TF10 = root mean squared, TF11 = total energy, TF13 = difference average, TF22 = dependence non uniformity, TF24 = dependence variance, TF25 = gray level non uniformity (gray-level dependence matrix), TF26 = large dependence emphasis, TF32 = run length non uniformity, TF35 = run variance, TF38 = size zone non uniformity normalized, TF40 = zone percentage. The data are expressed as means ± standard deviations.

Concurrently, cross-correlation analyses were conducted between the eGFR and the 40 texture features. The highest correlation coefficients were observed for two texture features (total energy [0.55, p < 0.001] and energy [0.55, p < 0.001]) in T1-weighted WO images. Table 8 shows the relationship between the eGFR and the 40 selected texture features in each imaging modality.

**Table 8:** The cross-correlation analyses between the eGFR and the 40 texture features derived from each imaging method.

Code	Feature name code	Imaging method									
		T1 IP		T1 OP		T1 WO		ADC map		T2* map	
		PCC	*p*-value	PCC	*p*-value	PCC	*p*-value	PCC	*p*-value	PCC	*p*-value
TF1	10th percentile	0.325	< 0.001	0.255	< 0.001	− 0.018	0.817	0.135	0.083	− 0.003	0.970
TF2	90th percentile	0.355	< 0.001	0.205	0.008	0.085	0.272	0.013	0.873	− 0.007	0.926
TF3	Energy	0.504	< 0.001	0.474	< 0.001	0.560	< 0.001	0.429	< 0.001	0.308	< 0.001
TF4	Entropy	− 0.004	0.955	− 0.078	0.314	0.172	0.027	− 0.207	0.008	0.009	0.909
TF5	Interquartile range	0.013	0.868	− 0.045	0.563	0.086	0.268	− 0.215	0.005	0.015	0.841
TF6	Mean absolute deviation	− 0.011	0.888	− 0.083	0.283	0.103	0.186	− 0.200	0.010	− 0.056	0.469
TF7	Mean	0.349	< 0.001	0.236	0.002	0.042	0.584	0.076	0.326	− 0.006	0.937
TF8	Median	0.346	< 0.001	0.208	0.007	0.026	0.738	0.086	0.268	0.029	0.706
TF9	Robust mean absolute deviation	0.005	0.948	− 0.050	0.520	0.085	0.271	− 0.207	0.007	0.008	0.915
TF10	Root mean squared	0.350	< 0.001	0.232	0.003	0.045	0.558	0.074	0.343	− 0.029	0.704
TF11	Total energy	0.503	< 0.001	0.471	< 0.001	0.558	< 0.001	0.072	0.353	0.413	< 0.001
TF12	Uniformity	− 0.042	0.591	0.021	0.786	− 0.207	0.007	0.251	0.001	− 0.065	0.400
TF13	Difference average	− 0.256	< 0.001	− 0.343	< 0.001	− 0.166	0.032	− 0.210	0.007	− 0.101	0.194
TF14	Difference entropy	− 0.271	< 0.001	− 0.348	< 0.001	− 0.159	0.040	− 0.212	0.006	− 0.091	0.243
TF15	Id	0.236	0.002	0.313	< 0.001	0.145	0.063	0.260	< 0.001	0.087	0.264
TF16	Idm	0.234	0.002	0.305	< 0.001	0.141	0.070	0.263	< 0.001	0.089	0.255
TF17	Inverse variance	0.255	< 0.001	0.308	< 0.001	0.143	0.067	0.213	0.006	0.056	0.469
TF18	Joint energy	0.034	0.667	0.093	0.230	− 0.129	0.098	0.281	< 0.001	− 0.038	0.626
TF19	Joint entropy	− 0.073	0.351	− 0.151	0.053	0.092	0.237	− 0.217	0.005	− 0.009	0.910
TF20	Maximum probability	− 0.039	0.612	− 0.005	0.945	− 0.149	0.055	0.284	< 0.001	− 0.058	0.455
TF21	Sum entropy	0.047	0.542	− 0.023	0.767	0.205	0.008	− 0.197	0.011	0.029	0.709
TF22	Dependence non uniformity	0.341	< 0.001	0.259	< 0.001	0.429	< 0.001	0.225	0.004	0.200	0.010
TF23	Dependence non uniformity normalized	− 0.216	0.005	− 0.328	< 0.001	− 0.189	0.015	− 0.263	< 0.001	− 0.053	0.496
TF24	Dependence variance	0.172	0.027	0.270	< 0.001	0.178	0.022	0.267	< 0.001	0.065	0.403
TF25	Gray level non uniformity	0.311	< 0.001	0.300	< 0.001	0.148	0.056	0.402	< 0.001	0.224	0.004
TF26	Large dependence emphasis	0.234	0.002	0.298	< 0.001	0.163	0.036	0.293	< 0.001	0.112	0.152
TF27	Small dependence emphasis	− 0.283	< 0.001	− 0.343	< 0.001	− 0.179	0.021	− 0.273	< 0.001	− 0.122	0.117
TF28	Gray level non uniformity	0.314	< 0.001	0.302	< 0.001	0.164	0.035	0.396	< 0.001	0.324	< 0.001
TF29	Gray level non uniformity normalized	− 0.027	0.727	0.028	0.714	− 0.200	0.010	0.227	0.003	− 0.031	0.691
TF30	Long run emphasis	0.235	0.002	0.305	< 0.001	0.161	0.039	0.288	< 0.001	0.088	0.260
TF31	Run entropy	0.095	0.223	0.021	0.787	0.263	< 0.001	− 0.056	0.472	0.130	0.094
TF32	Run length non uniformity	0.347	< 0.001	0.341	< 0.001	0.459	< 0.001	0.231	0.003	0.204	0.008
TF33	Run length non uniformity normalized	− 0.259	< 0.001	− 0.316	< 0.001	− 0.164	0.035	− 0.298	< 0.001	− 0.133	0.088
TF34	Run percentage	− 0.250	0.001	− 0.315	< 0.001	− 0.168	0.030	− 0.293	< 0.001	− 0.121	0.120
TF35	Run variance	0.221	0.004	0.302	< 0.001	0.165	0.033	0.285	< 0.001	0.070	0.369
TF36	Short run emphasis	− 0.257	< 0.001	− 0.312	< 0.001	− 0.158	0.041	− 0.299	< 0.001	− 0.136	0.081
TF37	Gray level non uniformity normalized	0.018	0.812	0.054	0.489	− 0.173	0.026	0.140	0.072	0.027	0.730
TF38	Size zone non uniformity normalized	− 0.326	< 0.001	− 0.361	< 0.001	− 0.184	0.017	− 0.246	0.001	− 0.146	0.061
TF39	Small area emphasis	− 0.332	< 0.001	− 0.359	< 0.001	− 0.179	0.021	− 0.246	0.001	− 0.156	0.044
TF40	Zone percentage	− 0.273	< 0.001	− 0.334	< 0.001	− 0.172	0.027	− 0.278	< 0.001	− 0.133	0.087

*ADC* apparent diffusion coefficient, *eGFR* estimated glomerular filtration rate, *GLCM* gray-level co-occurrence matrix, *GLDM* gray-level dependence matrix, *GLRLM* gray-level run length matrix, *GLSZM* gray-level size zone matrix, *IP* in-phase, *OP* opposed-phase, *PCC* Pearson's Correlation Coefficient, *TF* texture feature, *WO* water-only.

### Classification and validation

**Figure 1:**
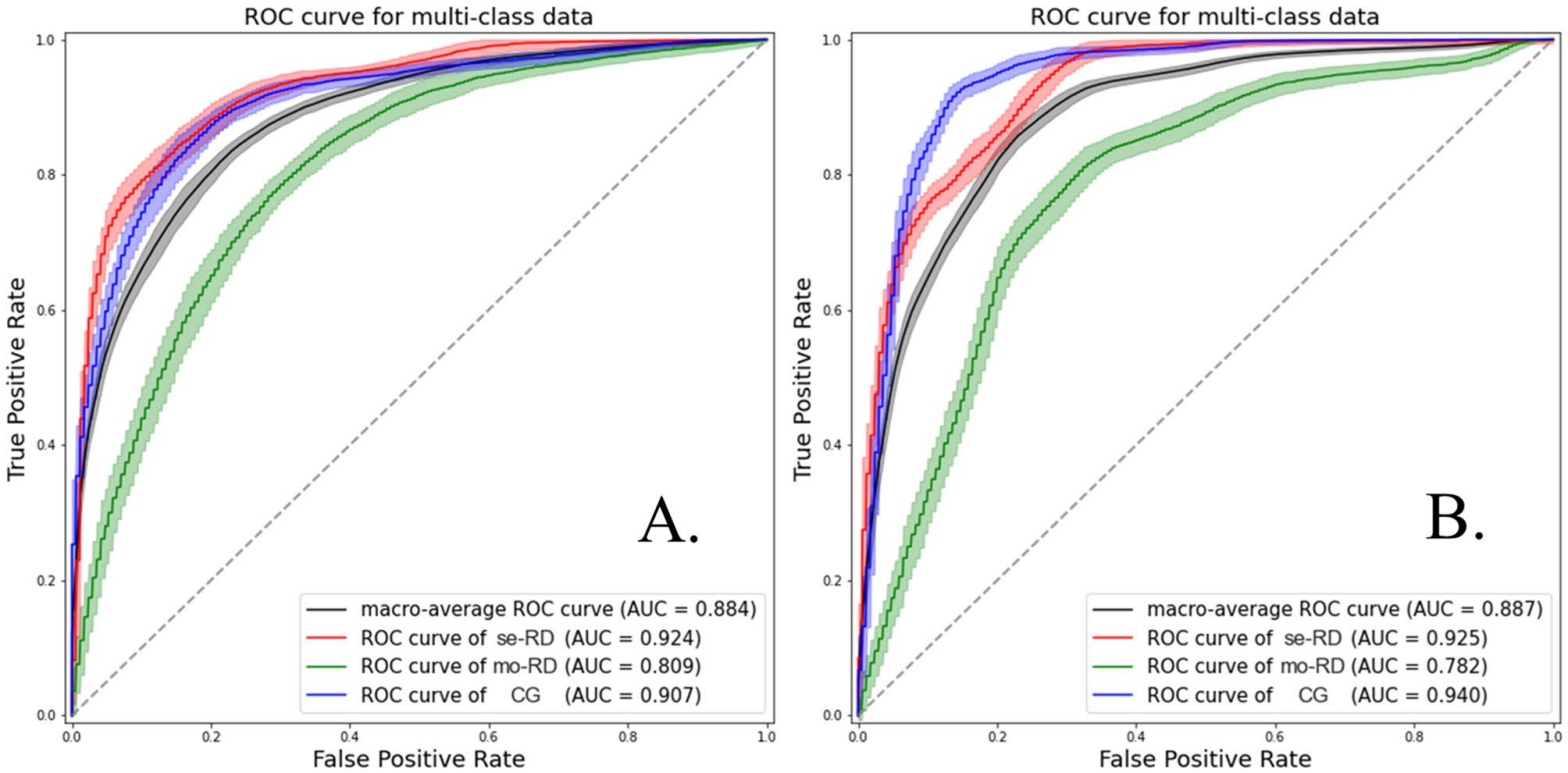
The receiver operating characteristic (ROC) curves and area under the curve (AUC) values of representative classification models using T1-weighted water-only images with a random forest classifier (**A**) and all T1-weighted images using a support vector machine with rbf kernel classifier (**B**) in classifying the three groups of chronic kidney disease. Severe renal dysfunction group (se-RD, estimated glomerular filtration rate [eGFR] < 30 mL/min/1.73 m^2^), moderate renal dysfunction group (mo-RD, 30 ≤ eGFR < 60 mL/min/1.73 m^2^), and control group (CG, eGFR ≥ 60 mL/min/1.73 m^2^). The AUC values are expressed as means.

**Figure 2:**
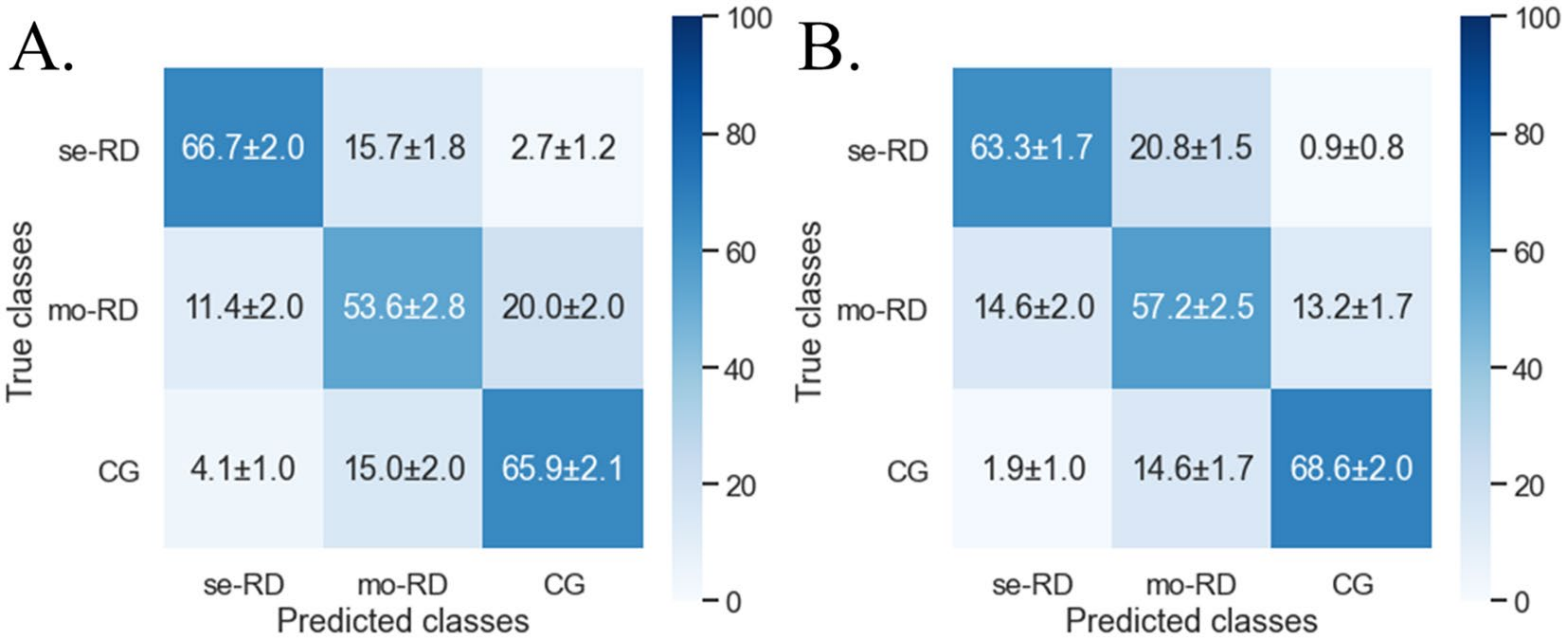
Confusion matrices show the status of representative classification models using T1-weighted water-only images with a random forest classifier (**A**) and all T1-weighted images using a support vector machine with rbf kernel classifier (**B**) in classifying the three groups of chronic kidney disease. Severe renal dysfunction group (se-RD, estimated glomerular filtration rate [eGFR] < 30 mL/min/1.73 m^2^), moderate renal dysfunction group (mo-RD, 30 ≤ eGFR < 60 mL/min/1.73 m^2^), and control group (CG, eGFR ≥ 60 mL/min/1.73 m^2^). The data are expressed as means ± standard deviations.

Receiver-operating characteristic (ROC) curve analyses were performed to compare the capacity of TA quantified from each imaging method to differentiate the three groups of CKD. Overall, the TA based on T1-weighted IP/OP/WO images provided better classification performance than that based on the ADC and T2* maps. Among the five imaging methods, the T1-weighted WO images obtained the highest classification scores: an accuracy of 76.5–82.0% and a macro-average area under the curve (AUC) of 0.812–0.884. Among the six classifiers we studied, the most favorable performance was observed in the random forest (RF) classifier. As for the support vector machine (SVM) classifier, the favorable results were obtained in the SVM with rbf kernel, whereas the results were poor in the SVM with sigmoid kernel. The results of all classification attempts are summarized in Tables 3, 4, 5, 6 and 7. Figures 1a and 2a present the ROC curve and the confusion matrix of the representative model (T1-weighted WO image with RF classifier). The confusion matrices and ROC curves of all classification attempts are summarized online in Supplementary Figs. S1–S5 and S8–S12, respectively.

### Combination models

The combination models derived from T1-weighted IP/OP/WO images (ALL T1WIs) and those derived from all imaging methods (ALL IMs) were evaluated. The selected texture features are listed in Tables 9 and 10. The best classification performance was observed in ALL T1WIs using the SVM with rbf kernel classifier: an accuracy of 82.8% and a macro-average AUC of 0.887. The results of all classification attempts are summarized in Tables 9 and 10. Figures 1b and 2b present the ROC curve and the confusion matrix of the representative model (ALL T1WIs using the SVM with rbf kernel classifier). The confusion matrices and ROC curves of all classification attempts are summarized online in Supplementary Figs. S6,S7 and S13,S14, respectively.

**Table 9:** Performance of each classification attempt in discriminating between the three groups in all T1-weighted imaging methods (ALL T1WIs).

	Accuracy (%)	Sensitivity (%)	Specificity (%)	AUC
**LDA (selected TFs = TF3 and TF19 derived from T1WI IP, TF3 from T1WI OP, and TF12 and TF31 from T1WI WO)**				
Macro-average	77.6 ± 0.9	66.5 ± 1.4	83.2 ± 0.8	0.844 ± 0.003
se-RD	81.0 ± 1.0	73.1 ± 1.6	84.9 ± 0.6	0.882 ± 0.003
mo-RD	68.7 ± 0.8	54.8 ± 1.5	75.6 ± 1.0	0.739 ± 0.006
CG	83.1 ± 1.0	71.1 ± 1.2	89.1 ± 0.8	0.897 ± 0.003
**SVM with linear kernel (selected TFs = TF3 and TF37 derived from T1WI IP, TF10 from T1WI OP, and TF3 and TF20 from T1WI WO)**				
Macro-average	81.5 ± 0.8	72.2 ± 1.4	86.1 ± 0.8	0.860 ± 0.004
se-RD	84.2 ± 0.8	73.4 ± 0.9	89.6 ± 0.9	0.878 ± 0.004
mo-RD	76.0 ± 0.7	67.3 ± 2.0	80.3 ± 0.8	0.794 ± 0.005
CG	84.3 ± 0.9	75.9 ± 1.3	88.5 ± 0.7	0.894 ± 0.004
**SVM with rbf kernel (selected TFs = TF3 and TF18 derived from T1WI IP, and TF18, TF20, and TF28 from T1WI WO)**				
Macro-average	82.8 ± 1.5	74.2 ± 2.4	87.1 ± 1.3	0.887 ± 0.006
se-RD	85.0 ± 1.6	74.5 ± 2.0	90.3 ± 1.3	0.925 ± 0.006
mo-RD	75.2 ± 1.3	67.3 ± 2.9	79.2 ± 1.4	0.782 ± 0.005
CG	88.1 ± 1.6	80.7 ± 2.4	91.8 ± 1.1	0.940 ± 0.006
**SVM with sigmoid kernel (selected TFs = TF25 derived from T1WI IP, TF3 and TF18 from T1WI OP, and TF4 and TF8 from T1WI WO)**				
Macro-average	77.3 ± 1.3	66.0 ± 2.4	83.0 ± 1.2	0.794 ± 0.006
se-RD	78.3 ± 1.3	58.7 ± 2.7	88.0 ± 1.2	0.797 ± 0.006
mo-RD	69.5 ± 1.1	55.4 ± 2.8	76.5 ± 1.5	0.696 ± 0.008
CG	84.3 ± 1.6	83.9 ± 1.6	84.5 ± 1.0	0.876 ± 0.008
**DT (selected TFs = TF3 and TF24 derived from T1WI IP, and TF28, TF31, and TF37 from T1WI OP)**				
Macro-average	75.3 ± 2.0	63.0 ± 4.0	81.5 ± 2.4	0.783 ± 0.013
se-RD	75.7 ± 1.9	65.3 ± 4.2	81.0 ± 2.7	0.816 ± 0.021
mo-RD	68.1 ± 1.8	54.3 ± 4.9	74.9 ± 2.8	0.688 ± 0.022
CG	82.2 ± 2.2	69.4 ± 3.0	88.6 ± 1.7	0.842 ± 0.017
**RF (selected TFs = TF3 derived from T1WI IP, TF3, TF13 and TF14 from T1WI OP, and TF2 from T1WI WO)**				
Macro-average	80.5 ± 1.6	70.8 ± 2.7	85.4 ± 1.4	0.874 ± 0.004
se-RD	82.0 ± 1.7	70.0 ± 2.6	88.0 ± 1.4	0.898 ± 0.004
mo-RD	74.3 ± 1.4	64.1 ± 3.3	79.4 ± 1.5	0.797 ± 0.008
CG	85.3 ± 1.8	78.3 ± 2.1	88.7 ± 1.2	0.916 ± 0.003

*AUC* area under the curve, *IP* in-phase, *OP* opposed-phase, *T1WI* T1-weighted imaging, *TF* texture feature, *WO* water-only, *DT* decision tree, *LDA* linear discriminant analysis, *SVM* support vector machine, *RF* random forest classifier, *se-RD* severe renal dysfunction (estimated glomerular filtration rate [eGFR] < 30 mL/min/1.73 m^2^, i.e., CKD stage G4–5), *mo-RD* moderate renal dysfunction (30 ≤ eGFR < 60 mL/min/1.73 m^2^, i.e., CKD stage G3a/3b), *CG* control group (eGFR ≥ 60 mL/min/1.73 m^2^, i.e., CKD stage G1–2). Feature name codes are as follows: TF2 = 90th percentile, TF3 = energy, TF4 = entropy, TF8 = median, TF10 = root mean squared, TF12 = uniformity, TF13 = difference average, TF14 = difference entropy, TF18 = joint energy, TF19 = joint entropy, TF20 = maximum probability, TF24 = dependence variance, TF25 = gray level non uniformity (gray-level dependence matrix), TF28 = gray level non uniformity (gray-level run length matrix), TF31 = run entropy, TF37 = gray level non uniformity normalized. The data are expressed as means ± standard deviations.

**Table 10:** Performance of each classification attempt in discriminating between the three groups in all imaging methods (ALL IMs).

	Accuracy (%)	Sensitivity (%)	Specificity (%)	AUC
**LDA (selected TFs = TF3 and TF19 derived from T1WI IP, TF3 from T1WI OP, and TF12 and TF31 from T1WI WO)**				
Macro-average	77.5 ± 0.8	66.2 ± 1.3	83.1 ± 0.7	0.832 ± 0.003
se-RD	78.6 ± 0.8	67.4 ± 1.3	84.2 ± 0.7	0.850 ± 0.003
mo-RD	70.1 ± 0.7	54.1 ± 1.3	78.2 ± 0.9	0.744 ± 0.007
CG	83.7 ± 0.8	77.2 ± 1.3	87.0 ± 0.6	0.890 ± 0.002
**SVM with linear kernel (selected TFs = TF3 and TF37 derived from T1WI IP, TF10 from T1WI OP, and TF3 and TF20 from T1WI WO)**				
Macro-average	81.9 ± 0.9	72.9 ± 1.7	86.5 ± 0.9	0.863 ± 0.003
se-RD	84.0 ± 1.0	79.9 ± 1.9	86.1 ± 0.9	0.885 ± 0.004
mo-RD	75.6 ± 0.8	58.9 ± 2.0	84.0 ± 0.9	0.781 ± 0.007
CG	86.2 ± 1.1	79.9 ± 1.1	89.3 ± 0.9	0.910 ± 0.004
SVM with rbf kernel (selected TFs = TF3 and TF18 derived from T1WI IP, TF18 and TF28 from T1WI WO, and TF20 from T2* map)				
Macro-average	81.6 ± 1.5	72.3 ± 2.9	86.2 ± 1.5	0.890 ± 0.005
se-RD	81.2 ± 1.5	71.7 ± 3.1	85.9 ± 1.7	0.911 ± 0.006
mo-RD	74.1 ± 1.3	60.0 ± 3.4	81.1 ± 1.8	0.798 ± 0.009
CG	89.4 ± 1.7	85.2 ± 2.2	91.5 ± 1.1	0.949 ± 0.006
SVM with sigmoid kernel (selected TFs = TF25 derived from T1WI IP, TF7 and TF11 from T1WI OP, TF21 from T1WI WO, and TF35 from T2* map)				
Macro-average	76.5 ± 1.4	64.7 ± 2.7	82.3 ± 1.5	0.822 ± 0.005
se-RD	77.4 ± 1.4	60.2 ± 2.6	86.0 ± 1.8	0.834 ± 0.005
mo-RD	67.9 ± 1.2	55.7 ± 3.6	73.9 ± 1.5	0.724 ± 0.008
CG	84.2 ± 1.6	78.2 ± 1.8	87.1 ± 1.2	0.897 ± 0.005
DT (selected TFs = TF3 and TF13 derived from T1WI IP, TF40 from T1WI OP, TF25 from T1WI WO, TF1 from T2* map)				
Macro-average	78.1 ± 1.9	67.1 ± 3.9	83.5 ± 2.1	0.806 ± 0.014
se-RD	81.7 ± 2.0	74.2 ± 3.7	85.5 ± 2.3	0.856 ± 0.020
mo-RD	69.9 ± 1.6	54.6 ± 4.8	77.5 ± 2.2	0.696 ± 0.024
CG	82.6 ± 2.1	72.5 ± 3.3	87.6 ± 1.9	0.864 ± 0.016
RF (selected TFs = TF3 derived from T1WI IP, TF3 from T1WI OP, TF18 from T2* map, and TF19 and TF40 from ADC map)				
Macro-average	81.3 ± 1.2	72.0 ± 2.2	86.0 ± 1.1	0.865 ± 0.004
se-RD	83.8 ± 1.2	70.9 ± 2.4	90.3 ± 1.1	0.866 ± 0.005
mo-RD	75.8 ± 1.1	63.6 ± 2.3	82.0 ± 1.3	0.815 ± 0.009
CG	84.4 ± 1.3	81.6 ± 2.0	85.8 ± 0.9	0.903 ± 0.004

*ADC* apparent diffusion coefficient, *AUC* area under the curve, *IP* in-phase, *OP* opposed-phase, *T1WI* T1-weighted imaging, *TF* texture feature, *WO* water-only, *DT* decision tree, *LDA* linear discriminant analysis, *SVM* support vector machine, *RF* random forest classifier, *se-RD* severe renal dysfunction (estimated glomerular filtration rate [eGFR] < 30 mL/min/1.73 m^2^, i.e., CKD stage G4–5), *mo-RD* moderate renal dysfunction (30 ≤ eGFR < 60 mL/min/1.73 m^2^, i.e., CKD stage G3a/3b), *CG* control group (eGFR ≥ 60 mL/min/1.73 m^2^, i.e., CKD stage G1–2). Feature name codes are as follows: TF1 = 10th percentile, TF3 = energy, TF7 = mean, TF10 = root mean squared, TF11 = total energy, TF12 = uniformity, TF13 = difference average, TF18 = joint energy, TF19 = joint entropy, TF20 = maximum probability, TF21 = sum entropy, TF25 = gray level non uniformity (gray-level dependence matrix), TF28 = gray level non uniformity (gray-level run length matrix), TF31 = run entropy, TF35 = run variance, TF37 = gray level non uniformity normalized, TF40 = zone percentage. The data are expressed as means ± standard deviations.

## Discussion

In the present study, we sought to investigate whether multiclass classification models based on TA of kidney MRI could predict the three eGFR groups of renal dysfunction. The results of our study suggest that TA of kidney MRI would be a modest predictor of these eGFR groups, but might not be a valuable differentiator in a clinical setting. TA quantified from T1-weighted IP/OP/WO images provided better classification performance compared with that of those based on ADC maps and T2* maps. Furthermore, we examined the combination models and showed that texture models derived from ALL T1WIs using the SVM with rbf kernel classifier afforded the moderate diagnostic performance as well. To our knowledge, this is the first study to evaluate the possibility of using multiclass models in the classification of eGFR groups. Several attempts have been made to differentiate between eGFR groups using TA of kidney MRI. Previous reports have shown the possibility of using TA based on DWI, BOLD, SWI, and T1 and T2 mapping^18,19^. In all these attempts, only binary classifiers were examined to differentiate between each eGFR group separately. However, in clinical practice, it is not uncommon for the classification of diseases to extend to three or more groups; therefore, it is reasonable to build multiclass problems for clinical use. According to the recent guidelines^2^, renal impairment and prognosis have been stratified into six disease groups based on the eGFR: G1 to G5, with G3 split into 3a and 3b. The most important cutoff points are eGFR 60 and 30 mL/min/1.73 m^2^, and previous studies on TA of kidney MRI were designed to classify the eGFR according to these cutoffs, except for one study that considered eGFR 90 mL/min/1.73 m^2^ as an additional cutoff point^19,22^. Therefore, our research also aimed to classify three groups, with cutoffs at eGFR 60 and 30 mL/min/1.73 m^2^. Ideally, the classification system of all six eGFR groups would be beneficial, although a large deviation in the distribution of each group prohibited these attempts.

Our study used Dixon-based T1-weighted images, which have not been fully discussed in the assessment of renal dysfunction. Dixon-based imaging, also called chemical shift imaging, uses the IP/OP cycling of fat and water molecules and allows the acquired images to be combined mathematically into four sequences: IP/OP/WO/fat-only (FO) images^23^. This technique has been used as a homogeneous fat suppression or fat quantification method in various medical imaging fields. The Dixon method has the potential for measuring the renal lipid accumulation in type II diabetes mellitus^11^. Moreover, the Dixon technique is useful in detecting iron deposition related to T2* effects and susceptibility artifacts owing to the double-echo sequence^24^. Additionally, Dixon-based images provide better signal-to-noise efficiency than other conventional fat-suppressed methods^25^. In our results, TA based on T1-weighted IP/OP/WO images demonstrated moderate classification scores, and the favorable classification scores were obtained by T1-weighted WO images. Although we could not analyze the FO image and fat fraction ratio map, as these were not available in all patients, a T1-weighted WO image can be regarded as a total fat-suppressed image and contains T1 information on components other than fat. In a recent study on T1 mapping, increased cortical T1 values and decreased T1 cortico-medullary differentiation correlated with the severity of renal impairment^20,21^. The changes in the T1 values could be attributed to renal physiological states, such as inflammation, hypoxia, and fibrosis^20,26–29^. In our opinion, T1-weighted WO images may represent these changes and could be useful in the non-invasive assessment of CKD etiologies.

In our study, TA based on the ADC and T2* maps showed relatively low diagnostic performance compared with that of those based on Dixon-based T1WI. In the ADC map, the texture features in the renal cortex had a good correlation with fibrosis and chronic lesions, and the texture features in the renal medulla were more related to renal function than those quantified from the renal cortex^19^. Additionally, the difference between the cortical and medullary ADC, the so-called delta-ADC, has been correlated with fibrosis in CKD^5,30^. However, since BOLD provides a marker of blood oxygenation levels, relative hypoxia associated with renal injury may be reflected by the T2* map. T2* measurement demonstrated a good correlation with the eGFR in patients with CKD, and TA of BOLD was linearly correlated with the eGFR in several studies^18,31–33^. In our results, TA of the ADC and T2* maps showed little correlation with the eGFR, and unsatisfactory results were obtained in multiclass problems. A possible explanation for the lower performances in the ADC and T2* maps is the difference in the quantification methods of texture features. We quantified the texture features from the renal parenchyma as a whole, whereas most other studies quantified the texture features from the renal cortex or medulla^5–10,19^. As described above, it would be favorable to consider the renal cortex and medulla separately for the assessment of the ADC and T2* maps. Moreover, our classification system was a multiclass model, and TA using non-linear classifiers based on clear images, such as Dixon-based T1WI, may be suitable for such systems. Regarding inter-reader reproducibility, the ICC values for the ADC and T2* maps were relatively low. These unsatisfactory results may be attributed to their relatively low resolution. Lower discriminative performance and reproducibility in TA of ADC maps have been reported due to the low resolution of the images^34,35^.

In our research, the texture features were extracted from the whole area of both kidneys, although in most studies, these were measured in the renal cortex and medulla separately, and in the ipsilateral kidney, mostly on the right side due to artifacts caused by factors such as intestinal gas, poor breath holds, and susceptibility effects^18^. Considering the different functionalities of the renal cortex and medulla, it might be more appropriate to assess them separately. However, in clinical practice, we contemplated that evaluating them as a whole would be simpler and easier to understand. For the ADC and T2* maps, in particular, the delineation between the renal cortex and medulla is difficult because of their relatively low resolution, and poor reproducibility is expected when considering the renal cortex and medulla separately. Moreover, in patients with advanced renal dysfunction, it is often difficult to distinguish between them because of cortical thinning^36,37^. Another point where our research differs from others is whether one or both sides of the kidney are considered. Our study evaluated both kidneys based on the idea that they might contain more integrated information concerning renal function. However, considering the time-consuming process of region of interest (ROI) delineation, it would be favorable to segment only one side of the kidney. If TA derived from one side of the kidney is sufficient, it would be beneficial to consider only one side of the kidney because of the severe artifacts on the other side. Moreover, in our study, we performed manual segmentation instead of using automatic methods. A method that automatically divides the renal parenchyma into 12 layers using a computer (twelve-layer concentric objects method) has been validated so far^32,38,39^. Its use may improve the discrimination capacity and reproducibility of our models, especially for the ADC and T2* maps, which need to be examined in the future.

In recent years, TA has become a promising technique for quantitative imaging analysis, providing biomarkers for pathological changes or the response to treatment^12–17^. In our analysis, TA of kidney MRI was not a good discriminator of eGFR groups, especially in the clinical setting. Texture features such as energy and total energy were frequently included in the selected features in most classification attempts, although their correlation with the eGFR was not good. It might be interesting to know the existence of universal texture parameters, as such features may represent the underlying pathophysiology of kidney disease. The energy and total energy show the magnitude of pixel values and accentuate the high signal intensities in the images^40^. In our opinion, cortico-medullary differentiation may have played a role in the renal dysfunction; as the renal function declined, decreased cortico-medullary differentiation was noted, as described above^20,21^. Both parameters showed decreased values in our results, implying decreased signal intensities in the whole area of the kidneys, and this may be affected by a decrease in the cortico-medullary difference. However, in most studies, entropy correlated well with the eGFR and showed the capacity to differentiate between the eGFR groups of patients with CKD^18,19,41^. Other reports state that skewness, kurtosis, and correlation may be useful in discriminating between these groups^18,19^. None of the studies commented on the energy and total energy. One reason for this discrepancy could be the difference in the classification system: a multiclass classification model was used in our study. Although we did not examine the binary classification for each border, it is suspected that the energy and total energy could be weak indicators for the overall classification of the eGFR groups. Another reason for the discrepancy is that we considered Dixon-based T1WI, whereas other studies mainly discussed DWI, BOLD, and SWI: the classifiers used in this study were mainly non-linear ML methods. In addition, TA was conducted on the whole region of the kidneys, whereas in other studies the renal cortex and medulla were analyzed separately. In this study, we also demonstrated the possible candidates of classifiers, such as SVM and RF. SVM has high generalizability since it can be used to select linear or non-linear kernels, and the 'rbf' (non-linear) kernel could be the most suited to our models. Generally, non-linear classifiers would show good performance in multiclass classification^42^, and our results showed this tendency as well.

Our study had many limitations. First, we retrospectively enrolled 166 patients from a single institution; this was a small sample with some imbalance between each group. A greater number of patients with more balanced grouping is needed to validate the results in the future. Second, since we excluded patients with renal lesions, some important renal diseases, such as polycystic kidney disease, were ignored in this analysis, which would have caused a selection bias. Third, the data were not divided into training and validation sets because of the limited number of patients; hence, further investigation using an external validation cohort should be performed in the future. Fourth, since we focused on classifications predicting the eGFR group, other important laboratory data or underlying pathologies were missed in this study. As stated above, renal lipid accumulation in diabetes mellitus can be assessed using the Dixon method^11^, which is worth investigating in the future. Fifth, we extracted texture features from one layer of the image as two-dimensional data; the use of only one layer may result in important texture features being missed. This problem can be solved by extracting three-dimensional features, although this approach may be time-consuming. Sixth, in this study, the individual texture feature sets were selected for each classifier and imaging method. Future studies should strictly compare the performances of classifiers or imaging methods by selecting one common feature set. Lastly, we should have examined other T1-weighted Dixon-based images, such as FO image and fat fraction ratio map, as well as other diffusion-based images such as intra-voxel incoherent motion.

In conclusion, multiclass classification models based on TA of kidney MRI showed modest classification performance for predicting the eGFR in patients with CKD. TA based on Dixon-based T1WI, particularly WO images, showed moderate performance. Energy and total energy were weakly correlated with the eGFR. Our results were limited in terms of the clinical value of TA of kidney MRI, and thus further studies should verify its reproducibility and feasibility.

## Methods

### Subjects

This study was approved by the Research Ethics Committee of the Saitama Medical University Hospital. The requirement for informed consent was waived by the committee (approval number BYOU2022-037). All experiments were performed in accordance with the relevant guidelines and regulations.

**Figure 3:**
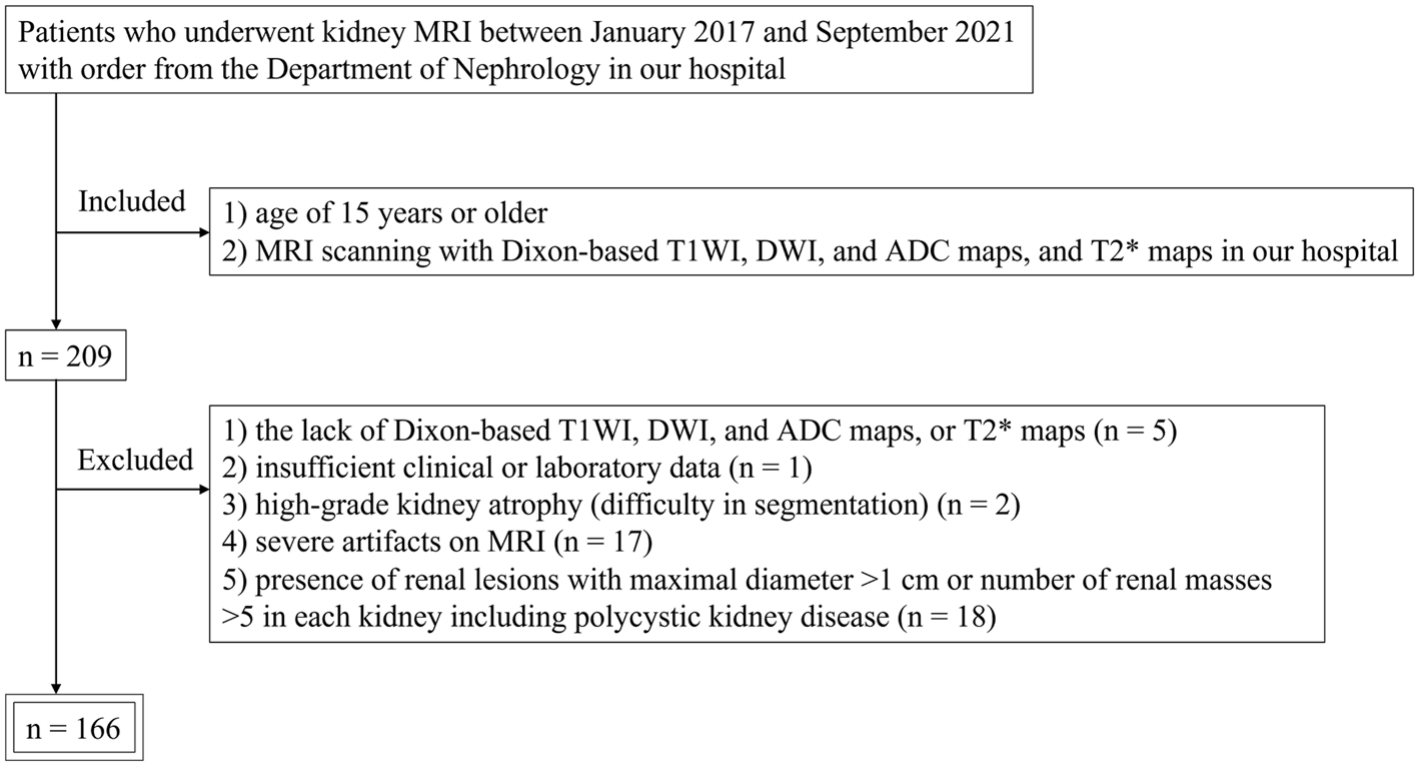
Flow chart of the inclusion and exclusion criteria for the study. *ADC* apparent diffusion coefficient, *DWI* diffusion-weighted imaging, *MRI* magnetic resonance imaging, *T1WI* T1-weighted imaging.

Figure 3 presents the inclusion and exclusion criteria for this study. We identified and reviewed 209 patients referred from the Department of Nephrology in our hospital who underwent kidney MRI between January 2017 and September 2021. The inclusion criteria included: (1) age of 15 years or older; and (2) MRI scanning with Dixon-based T1WI, DWI, and ADC maps and T2* maps in our hospital. The exclusion criteria included: (1) lack of Dixon-based T1WI, DWI, and ADC maps or T2* maps (n = 5); (2) insufficient clinical or laboratory data (n = 1); (3) high-grade kidney atrophy (difficulty in segmentation) (n = 2); (4) severe artifacts on MRI (n = 17); and (5) presence of renal lesions with maximal diameter > 1 cm or number of renal masses > 5 in each kidney, including polycystic kidney disease (n = 18). In total, 166 patients were enrolled.

The eGFR was calculated using Eq. (1):

1 $${\text{eGFR }} ( {{\text{mL}}/{\text{min}}/{1}.{\text{73 m}}^{{2}} } ) = { 194 } \times {\text{ sCr}}^{{ - 1.094}} \times {\text{ age}}^{{ - 0.{287}}} \times 0.739\, ( {\text{for women}} ),$$

where age is in years and serum creatinine (sCr) is in mg/dL. The eGFR was defined as 120 mL/min/1.73 m^2^ if it was greater than 120 mL/min/1.73 m^2^ as calculated using Eq. (1).

The patients were divided into three groups according to the eGFR: se-RD group (eGFR < 30 mL/min/1.73 m^2^, i.e., CKD stage G4–5), mo-RD group (30 ≤ eGFR < 60 mL/min/1.73 m^2^, i.e., CKD stage G3a/b), and CG (eGFR ≥ 60 mL/min/1.73 m^2^, i.e., CKD stage G1–2).

### MRI acquisition

MRI images were acquired using a 3.0 Tesla superconducting unit (Skyra, Siemens Healthcare, Erlangen, Germany) with a spine coil and an 18-channel phased-array body coil. The standard dedicated MRI protocol consisted of the following sequences: Dixon-based T1WI with the 3D gradient-echo method, DWI with multiple b-factors, and T2*WI with multiple gradient-echoes obtained in the coronal plane. For the Dixon-based T1WI, only IP/OP/WO images were used in the analysis, as other images (such as fat-only images and fat fraction ratio maps) were not generated in all patients. The ADC map was automatically generated based on a monoexponential fitting model using DWI at the four b-factors. In BOLD, 12 T2* WIs corresponding to 12 different gradient echoes were acquired. T2* maps were generated on a pixel-by-pixel basis by fitting a linear regression method through the logarithms of the signal intensities versus their 12 echo times.

Table 11 presents the representative MRI scanning sequences and parameters.

**Table 11:** Representative MRI scanning sequences and parameters.

Parameters	T1WI IP/OP/WO	DWI/ADC map	BOLD (T2* map)
TR (ms)	5.35	1100	175
TE (ms)	2.46, 3.69	70	4.92, 7.38, 9.84, 12.30, 14.76, 17.22, 19.68, 22.14, 24.60, 27.06, 29.52, and 31.98
FA (°)	10	N/A	50
FOV (mm)	360 × 360 × 144	360 × 360 × 45	360 × 360 × 27
Voxel size (mm)	1.1 × 1.1 × 3.0	1.4 × 1.4 × 3.0	1.4 × 1.4 × 5.0
Recon matrix	320	128	256
Slice thickness (mm)	3	3	5
b value (mm^2^/s)		0, 200, 400, 600	
Respiratory compensation	Breath hold	Free breathing	Breath hold

*ADC* apparent diffusion coefficient, *BOLD* blood oxygenation level-dependent imaging, *DWI* diffusion-weighted imaging, *FA* flip angle, *FOV* field of view, *IP* in-phase, *OP* opposed-phase, *T1WI* T1-weighted imaging, *TE* echo time, *TR* repetition time, *WO* water-only.

### Data analysis procedures

**Figure 4:**
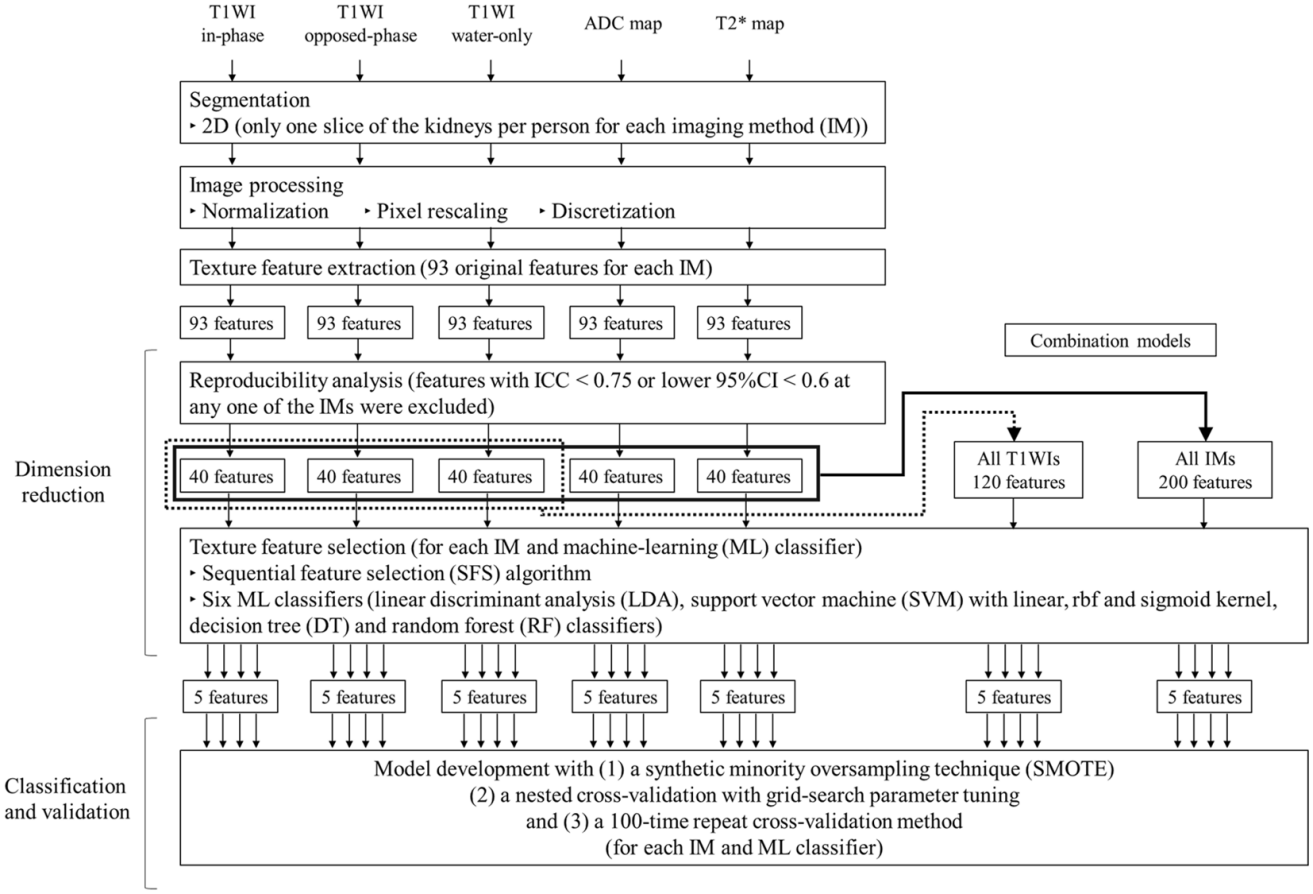
Flow chart showing the technical study pipeline. After segmentation, image processing, texture feature extraction, and reproducibility analysis were conducted for each imaging method (T1-weighted in-phase/opposed-phase/water-only images, ADC maps, and T2* maps), followed by texture feature selection and ML-based model construction in separate classification attempts. The combinations of texture features were also examined: those derived from all T1-weighted images and those derived from all imaging methods.

Figure 4 presents the data analysis workflow. After segmentation, image processing, texture feature extraction, and reproducibility analysis were performed for each imaging method (T1-weighted IP/OP/WO images, ADC map, and T2* map), followed by texture feature selection and ML-based model construction in separate classification attempts. The combinations of the texture features were also examined: those derived from all T1-weighted images (ALL T1WIs) and those derived from all imaging methods (ALL IMs).

### Texture feature extraction

**Figure 5:**
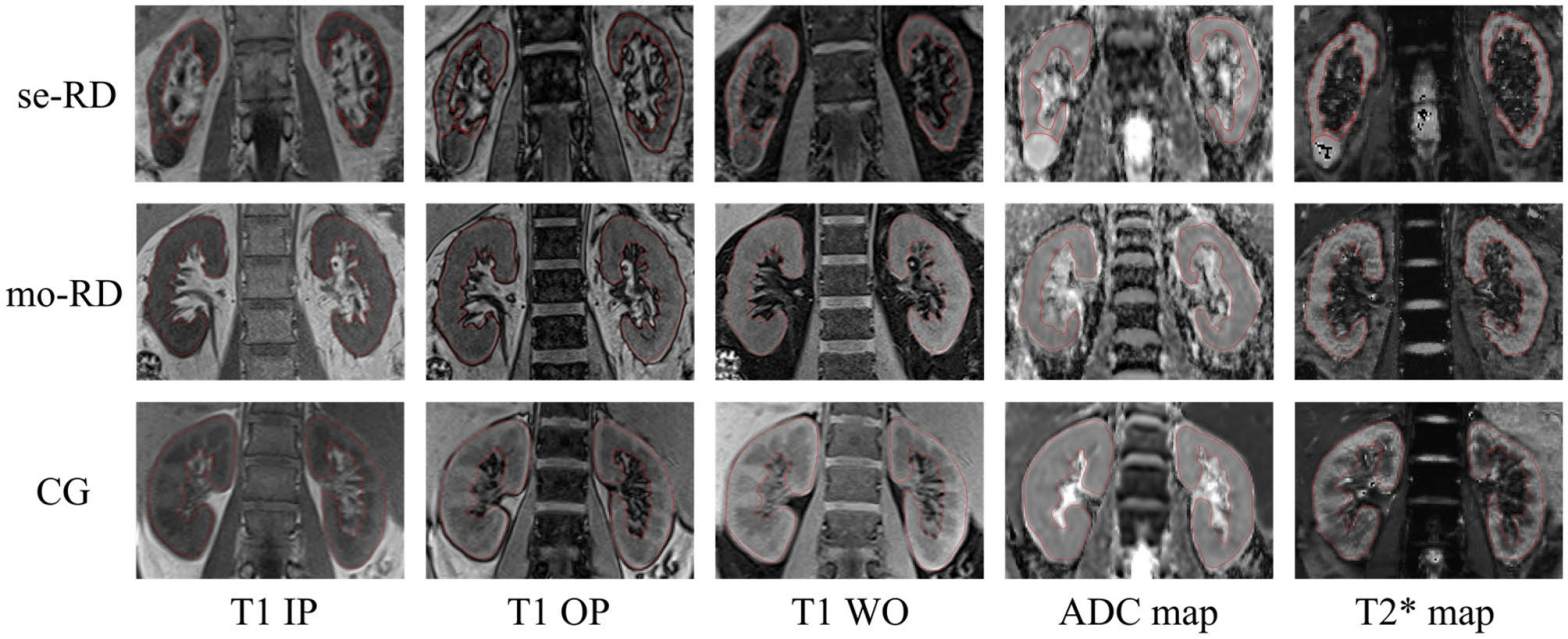
A method to set the region of interest (ROI) for each group and each image. ROIs were manually drawn on the contour lines of both kidneys, as shown by the red curves (avoiding the cystic area). *ADC* apparent diffusion coefficient, *IP* in-phase, *OP* opposed-phase, *WO* water-only. Severe renal dysfunction group (se-RD, estimated glomerular filtration rate [eGFR] < 30 mL/min/1.73 m^2^), moderate renal dysfunction group (mo-RD, 30 ≤ eGFR < 60 mL/min/1.73 m^2^), and control group (CG, eGFR ≥ 60 mL/min/1.73 m^2^).

Segmentation was performed using an open-source software (ITK-SNAP version 3.8.0). One slice of T1-weighted IP/OP/WO images, ADC maps, and T2* map images in the coronal plane were selected for each patient. An irregular two-dimensional ROI was drawn manually to contain the outline borders of the entire region of both kidneys on each selected image, and the cystic region was avoided to the maximum extent (Fig. 5). Two radiologists with 7 and 6 years of experience performed ROI delineation independently to assess the inter-observer reproducibility in the segmentation process. Both radiologists were blinded to the clinical information.

To avoid data heterogeneity bias, all MRI data were subjected to image normalization (the intensity of the image was scaled to 0–100) and resampled to the same resolution (3 × 3 × 3 mm) before feature extraction. The texture features were calculated using an open-source software package capable of extracting a large panel of engineered features from medical images (PyRadiomics version 2.1.0). Texture features were calculated based on six feature classes (first-order statistics, gray-level co-occurrence matrix, gray-level dependence matrix, gray-level run-length matrix, gray-level size zone matrix, and neighboring gray-tone difference matrix). Ninety-three texture features were extracted and analyzed to select the most valuable features for discerning the three CKD groups with each imaging method.

### Dimension reduction of texture features

We performed dimension reduction of texture features to avoid overfitting and generalization errors in the classification models. After normalizing the numeric values as z-scores, the ICC was measured to evaluate the inter-observer reproducibility. Features with poor reproducibility (ICC < 0.75 or lower 95% CI < 0.6) in any of the imaging methods were excluded. Furthermore, the SFS algorithm, a wrapper-based greedy search algorithm, was used for feature selection. This algorithm identifies feature subsets that maximize the performance of predictive models by adding or eliminating features stepwise based on a user-defined classifier algorithm. We considered four representative ML classifiers in this study: linear discriminant analysis (LDA), SVM, decision tree (DT), and RF classifiers. As for the SVM algorithm, various kernel functions provide different decision-making algorithms and generate versatility. We adopted three representative kernels separately and compared their results in this study: linear, rbf, and sigmoid kernels. Thus, in total, we tested six different ML classifiers: LDA; SVM with linear, rbf, and sigmoid kernels; DT; and RF. By using the SFS algorithm, a subset of features that provided the best classification accuracies in each ML classifier was selected. The number of texture features was reduced to five in this step to prevent overfitting due to the small sample size.

Concurrently, the relationship between the eGFR and the selected texture features for each imaging modality was examined using Pearson’s correlation coefficient.

### Classification and validation

Multiclass classification models were created using the six ML classifiers described above and validated using the cross-validation method. We adopted the following methods to obtain the generalizability of our classification models and to test their applicability: (1) synthetic minority oversampling technique (SMOTE), (2) nested cross-validation with grid-search parameter tuning, and (3) 100-time repeat cross-validation method.

Since our data had an imbalance between classes, we applied a SMOTE method before the final classification and validation step. This method creates synthetic instances that are not exact replications and increases the datasets of the minority group without damaging the structure of the actual data^43–45^. We applied this technique to augment the minority group datasets (i.e., 45 control cases and 36 severe RD cases), while preserving the majority group datasets (i.e., 85 moderate RD cases), resulting in 85 labeled cases for each class (255 cases in total).

Several intrinsic hyperparameters are known for the SVM, DT, and RF classifiers, and the classification performance could be changed by attenuating these values. Thus, a nested cross-validation method with tenfold inner loops and tenfold outer loops was adopted to tune the parameters of these classifiers to avoid the double-dipping phenomenon, a potential bias^46,47^, which indicates that training and test data were used for feature selection and model development, along with validation. A grid-search system was used for parameter tuning, in which a set of parameters with a discrete number of values was tested repeatedly to obtain the best parameter combination. The following hyperparameters were tested: C-value = 1, 10, and 100 and gamma = 0.001, 0.01, and 0.1 for SVM; and max-depth = 2, 4, and 6 and min-samples-leaf = 0.1, 0.5, 1, 5, and 10 for DT and RF.

The cross-validation method was repeated 100 times to ensure the stability and reproducibility of our results. We repeated a SMOTE process along with a nested cross-validation as data augmented by the SMOTE may have some arbitrariness. The performance of the classifiers was evaluated using ROC curve analysis and the AUC. The accuracy, sensitivity, and specificity for each group and macro-average of all groups were calculated based on the confusion matrix of the classification results.

### Combination models

We also evaluated the classification performance of the combination models derived from ALL T1WIs and those derived from ALL IMs. The SFS algorithm was used again for feature selection, and the number of texture features was reduced to 5. The multiclass classification models were created using the six ML classifiers mentioned above, and the performance of the classifiers was evaluated in the same manner as described above.

Statistical analyses were performed using an open-source software package (Python scikit-learn 0.22.1). Statistical significance was set at P < 0.05.

## Supplementary Information

Supplementary Information 1.

Supplementary Information 2.

Supplementary Information 3.

Supplementary Information 4.

Supplementary Information 5.

Supplementary Information 6.

## Data Availability

The authors declare that all data supporting the findings of this study are available within the article.

## Abbreviations

ADC: Apparent diffusion coefficient
AUC: Area under the curve
BOLD: Blood oxygenation level-dependent imaging
CKD: Chronic kidney disease
CG: Control group
DT: Decision tree
DWI: Diffusion-weighted imaging
eGFR: Estimated glomerular filtration rate
ICC: Intraclass correlation coefficient
IP: In-phase
LDA: Linear discriminant analysis
ML: Machine learning
mo-RD: Moderate renal dysfunction
MRI: Magnetic resonance imaging
OP: Opposed-phase
RF: Random forest
ROC: Receiver operating characteristic
ROI: Region of interest
se-RD: Severe renal dysfunction
SFS: Sequential feature selection
SVM: Support vector machine
SWI: Susceptibility-weighted imaging
T1WI: T1-weighted imaging
T2*WI: T2*-weighted imaging
TA: Texture analysis
WO: Water-only
