# Supplementary material for: The utility of texture analysis of kidney MRI for evaluating renal dysfunction with multiclass classification model
Source: Sci Rep. 2022 Aug 30;12:14776. doi: 10.1038/s41598-022-19009-7 (PMC9427930; doi:10.1038/s41598-022-19009-7)
Supplement: Supplementary file 6 — Supplementary Information 6. [file 41598_2022_19009_MOESM6_ESM.docx]

The Utility of Texture Analysis of Kidney MRI for Evaluating Renal Dysfunction with Multiclass Classification Model

**Names of the Authors:**

Yuki Hara^1^ , Keita Nagawa^1^ , Yuya Yamamoto^1^ , Kaiji Inoue^1^ , Kazuto Funakoshi^1^ , Tsutomu Inoue^2^ , Hirokazu Okada^2^ , Masahiro Ishikawa^3^ , Naoki Kobayashi^3^ , Eito Kozawa^1^


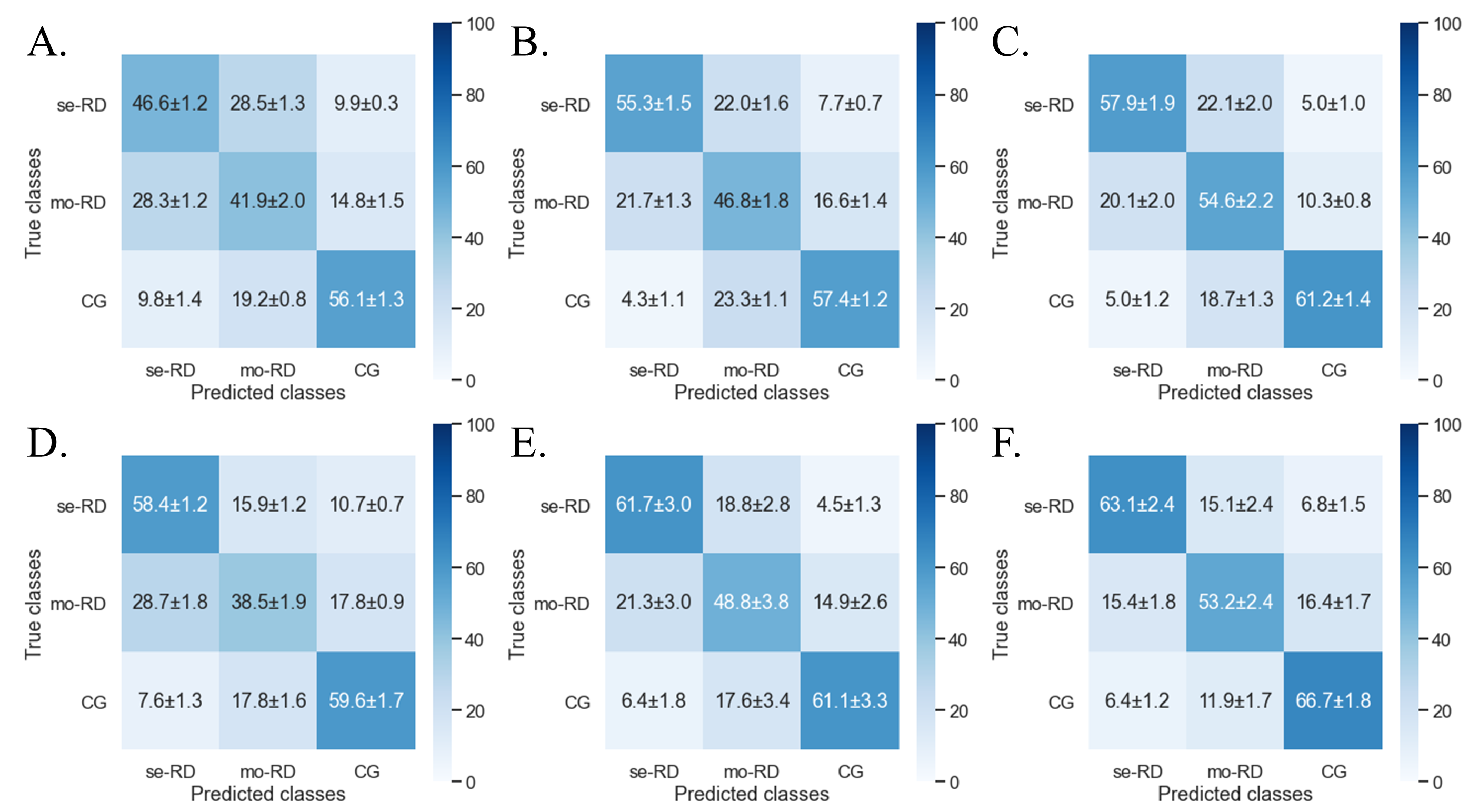


Supplementary Figure S1. Confusion matrices show the status of multiclass classifications using T1-weighted in-phase (IP) image with linear discriminant analysis (LDA) (**A**); support vector machine (SVM) with linear (**B**), rbf (**C**) and sigmoid (**D**) kernels; decision tree (DT) (**E**); and random forest (RF) (**F**) classifiers, in classifying the three groups of chronic kidney disease. Severe renal dysfunction group (se-RD, eGFR < 30 mL/min/1.73 m²), moderate renal dysfunction group (mo-RD, eGFR ≥ 30 and < 60 mL/min/1.73 m^2^), and control group (CG, eGFR ≥ 60 mL/min/1.73 m^2^). The data are expressed as means ± standard deviations.


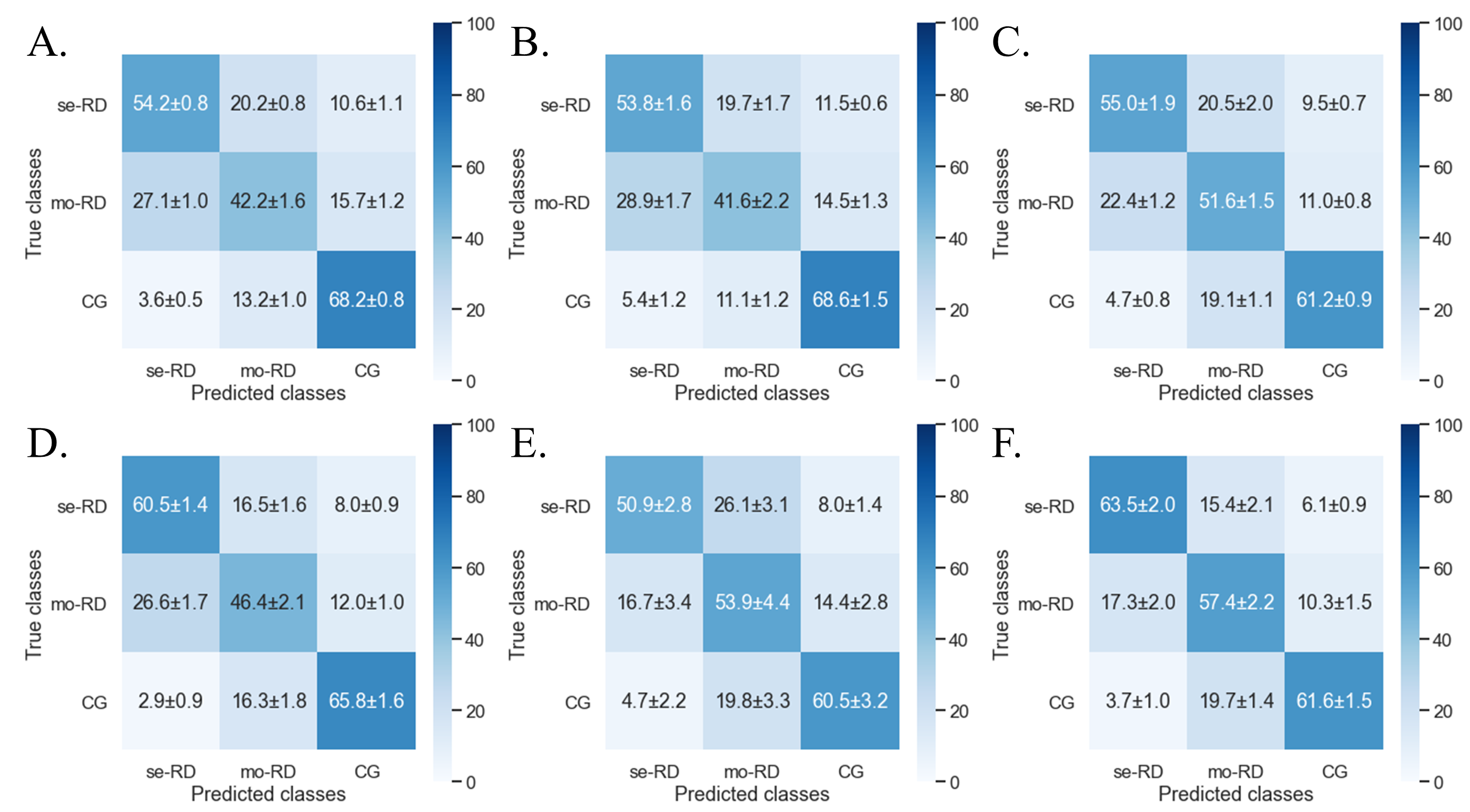
Supplementary Figure S2. Confusion matrices show the status of multiclass classifications using T1-weighted opposed-phase (OP) image with linear discriminant analysis (LDA) (**A**); support vector machine (SVM) with linear (**B**), rbf (**C**) and sigmoid (**D**) kernels; decision tree (DT) (**E**); and random forest (RF) (**F**) classifiers, in classifying the three groups of chronic kidney disease. Severe renal dysfunction group (se-RD, eGFR < 30 mL/min/1.73 m²), moderate renal dysfunction group (mo-RD, eGFR ≥ 30 and < 60 mL/min/1.73 m^2^), and control group (CG, eGFR ≥ 60 mL/min/1.73 m^2^). The data are expressed as means ± standard deviations.


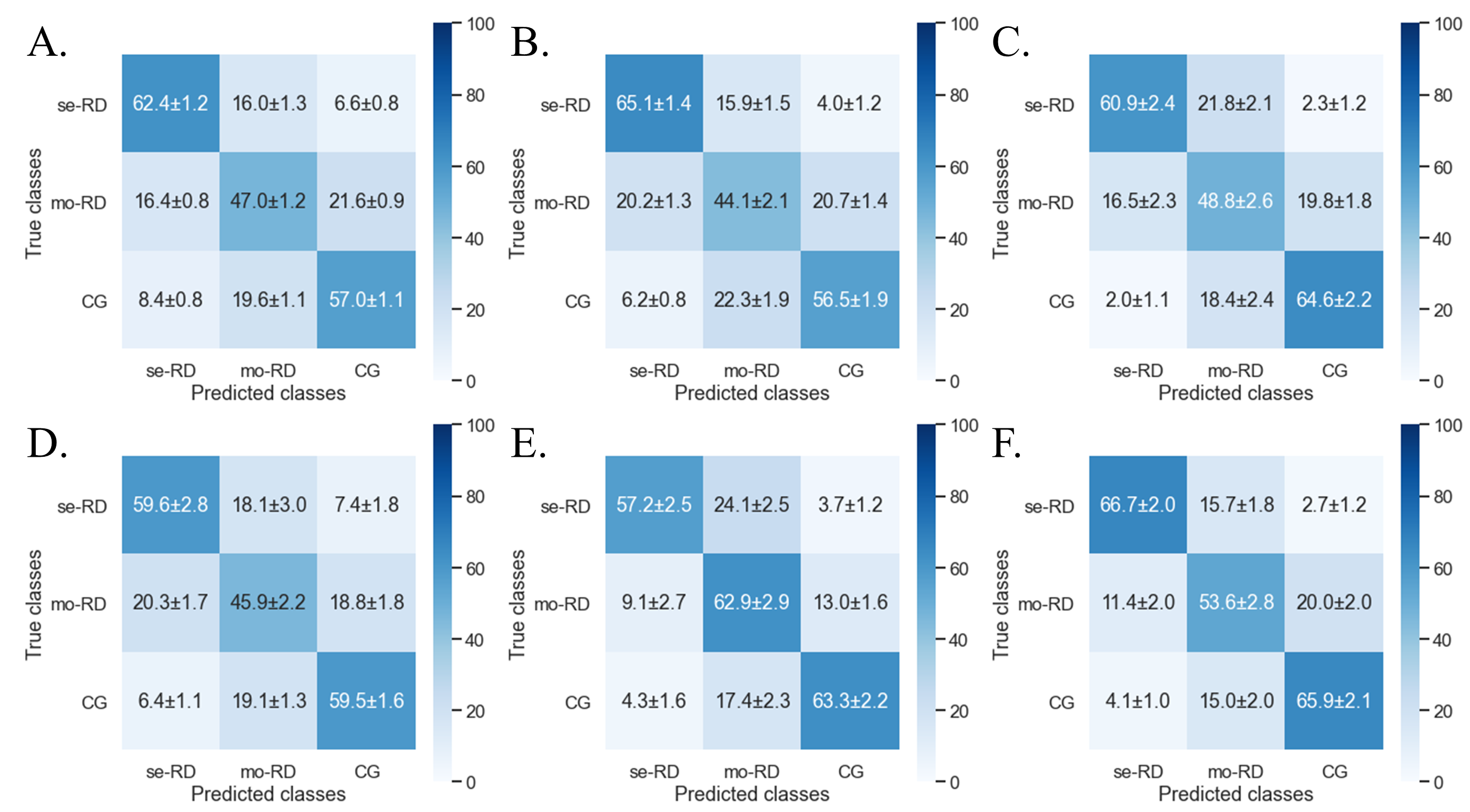
Supplementary Figure S3. Confusion matrices show the status of multiclass classifications using T1-weighted water-only (WO) image with linear discriminant analysis (LDA) (**A**); support vector machine (SVM) with linear (**B**), rbf (**C**) and sigmoid (**D**) kernels; decision tree (DT) (**E**); and random forest (RF) (**F**) classifiers, in classifying the three groups of chronic kidney disease. Severe renal dysfunction group (se-RD, eGFR < 30 mL/min/1.73 m²), moderate renal dysfunction group (mo-RD, eGFR ≥ 30 and < 60 mL/min/1.73 m^2^), and control group (CG, eGFR ≥ 60 mL/min/1.73 m^2^). The data are expressed as means ± standard deviations.


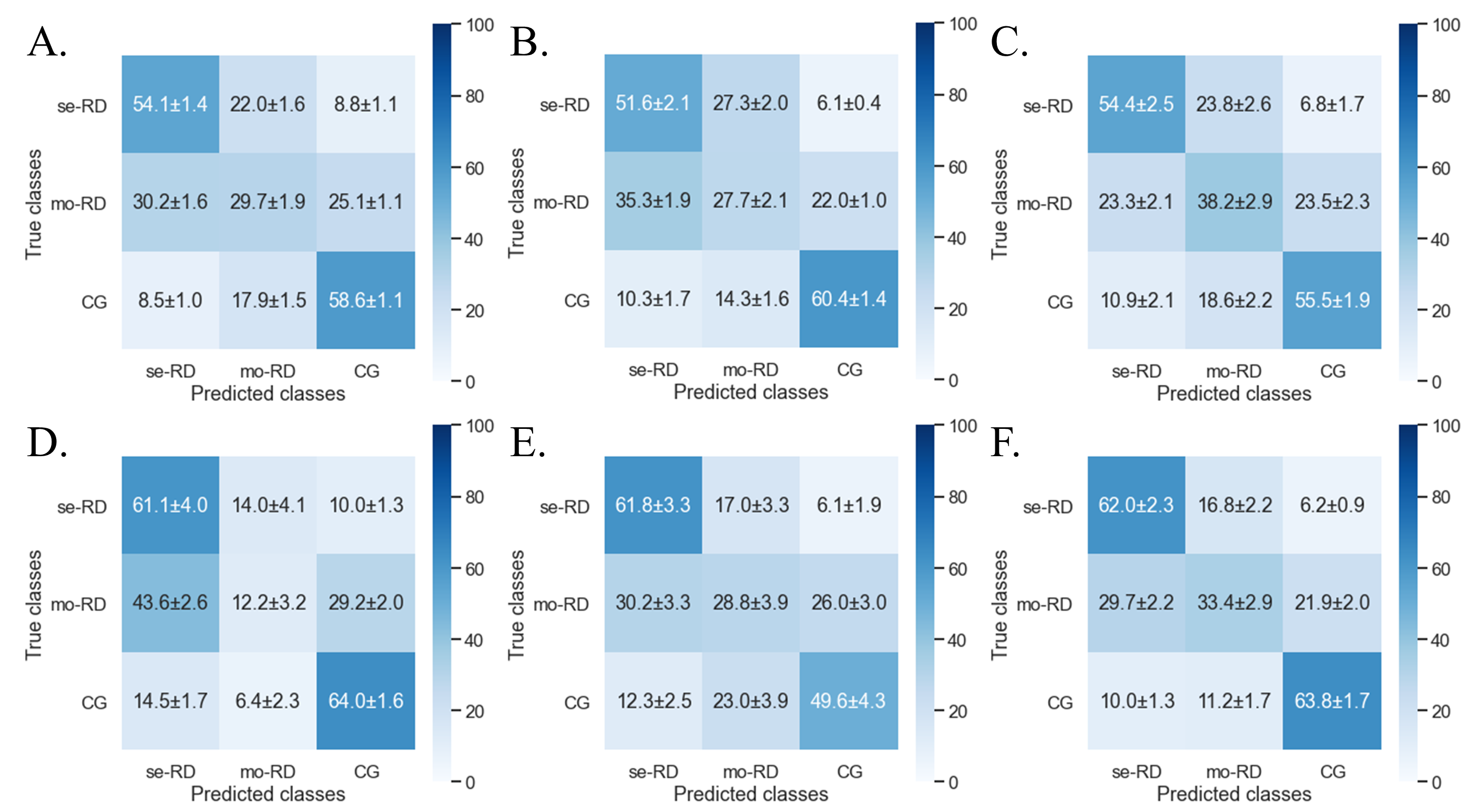
Supplementary Figure S4. Confusion matrices show the status of multiclass classifications using apparent diffusion coefficient (ADC) map with linear discriminant analysis (LDA) (**A**); support vector machine (SVM) with linear (**B**), rbf (**C**) and sigmoid (**D**) kernels; decision tree (DT) (**E**); and random forest (RF) (**F**) classifiers, in classifying the three groups of chronic kidney disease. Severe renal dysfunction group (se-RD, eGFR < 30 mL/min/1.73 m²), moderate renal dysfunction group (mo-RD, eGFR ≥ 30 and < 60 mL/min/1.73 m^2^), and control group (CG, eGFR ≥ 60 mL/min/1.73 m^2^). The data are expressed as means ± standard deviations.


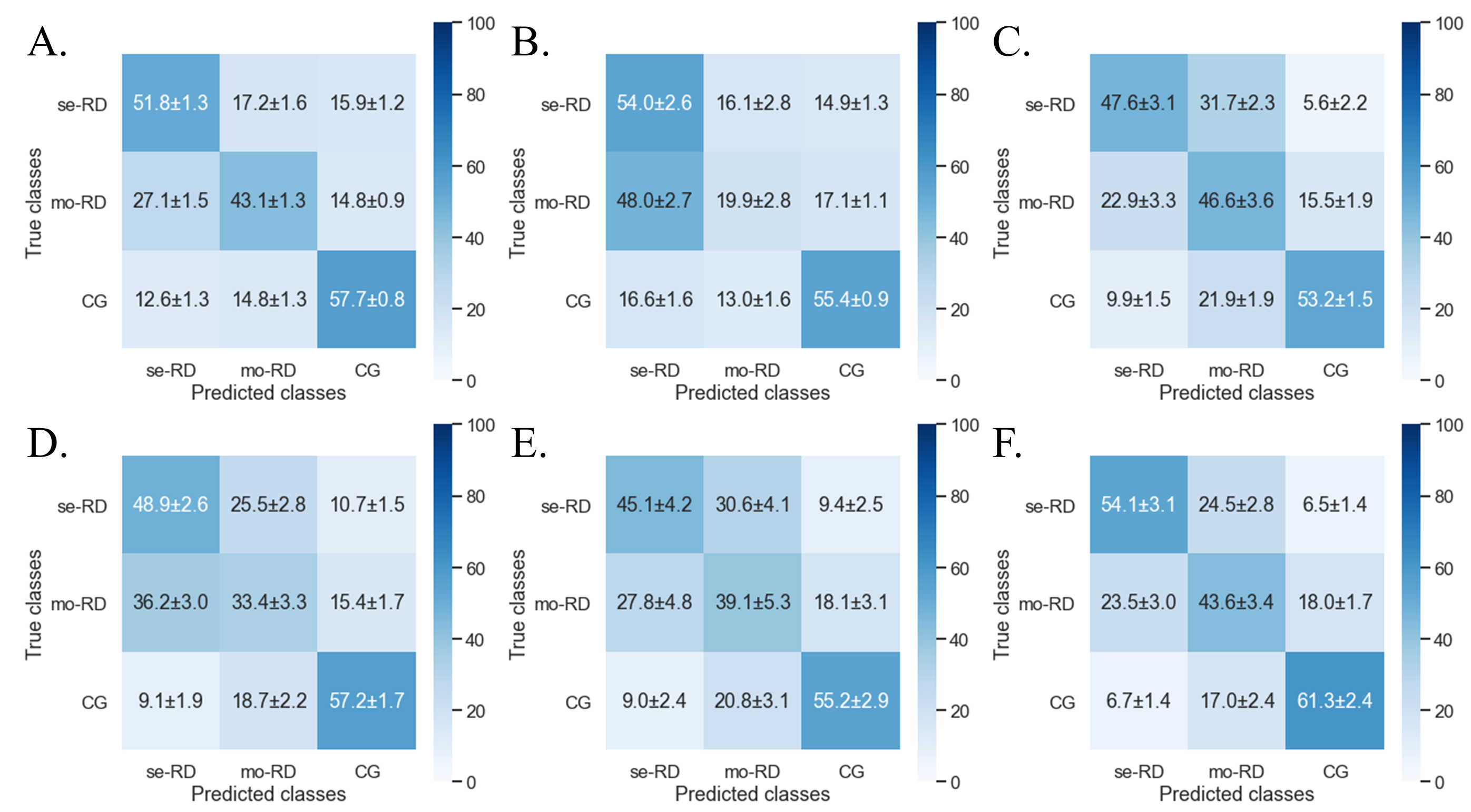


Supplementary Figure S5. Confusion matrices show the status of multiclass classifications using T2* map with linear discriminant analysis (LDA) (**A**); support vector machine (SVM) with linear (**B**), rbf (**C**) and sigmoid (**D**) kernels; decision tree (DT) (**E**); and random forest (RF) (**F**) classifiers, in classifying the three groups of chronic kidney disease. Severe renal dysfunction group (se-RD, eGFR < 30 mL/min/1.73 m²), moderate renal dysfunction group (mo-RD, eGFR ≥ 30 and < 60 mL/min/1.73 m^2^), and control group (CG, eGFR ≥ 60 mL/min/1.73 m^2^). The data are expressed as means ± standard deviations.


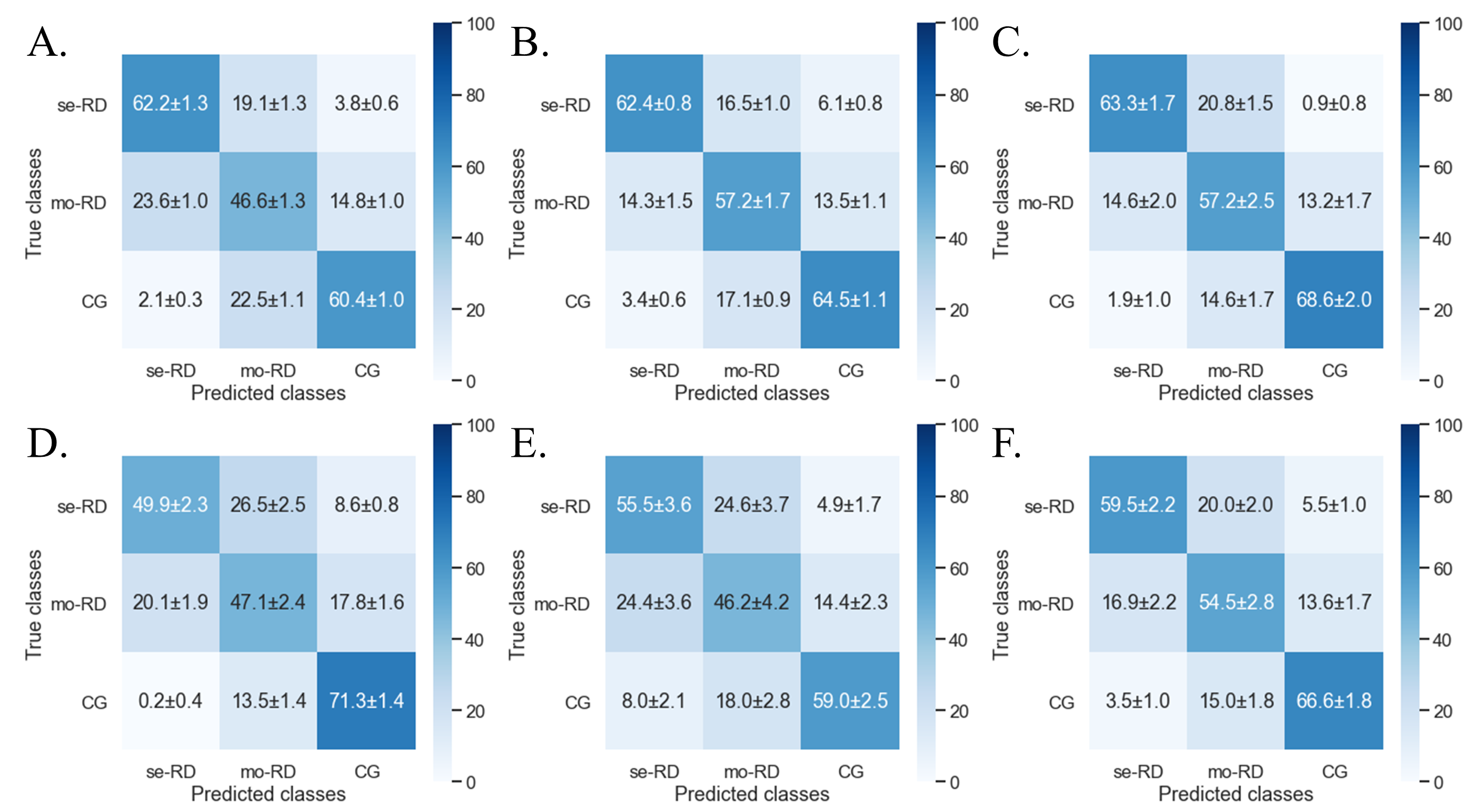


Supplementary Figure S6. Confusion matrices show the status of multiclass classifications using all T1-weighted images (ALL T1WIs) with linear discriminant analysis (LDA) (**A**); support vector machine (SVM) with linear (**B**), rbf (**C**) and sigmoid (**D**) kernels; decision tree (DT) (**E**); and random forest (RF) (**F**) classifiers, in classifying the three groups of chronic kidney disease. Severe renal dysfunction group (se-RD, eGFR < 30 mL/min/1.73 m²), moderate renal dysfunction group (mo-RD, eGFR ≥ 30 and < 60 mL/min/1.73 m^2^), and control group (CG, eGFR ≥ 60 mL/min/1.73 m^2^). The data are expressed as means ± standard deviations.


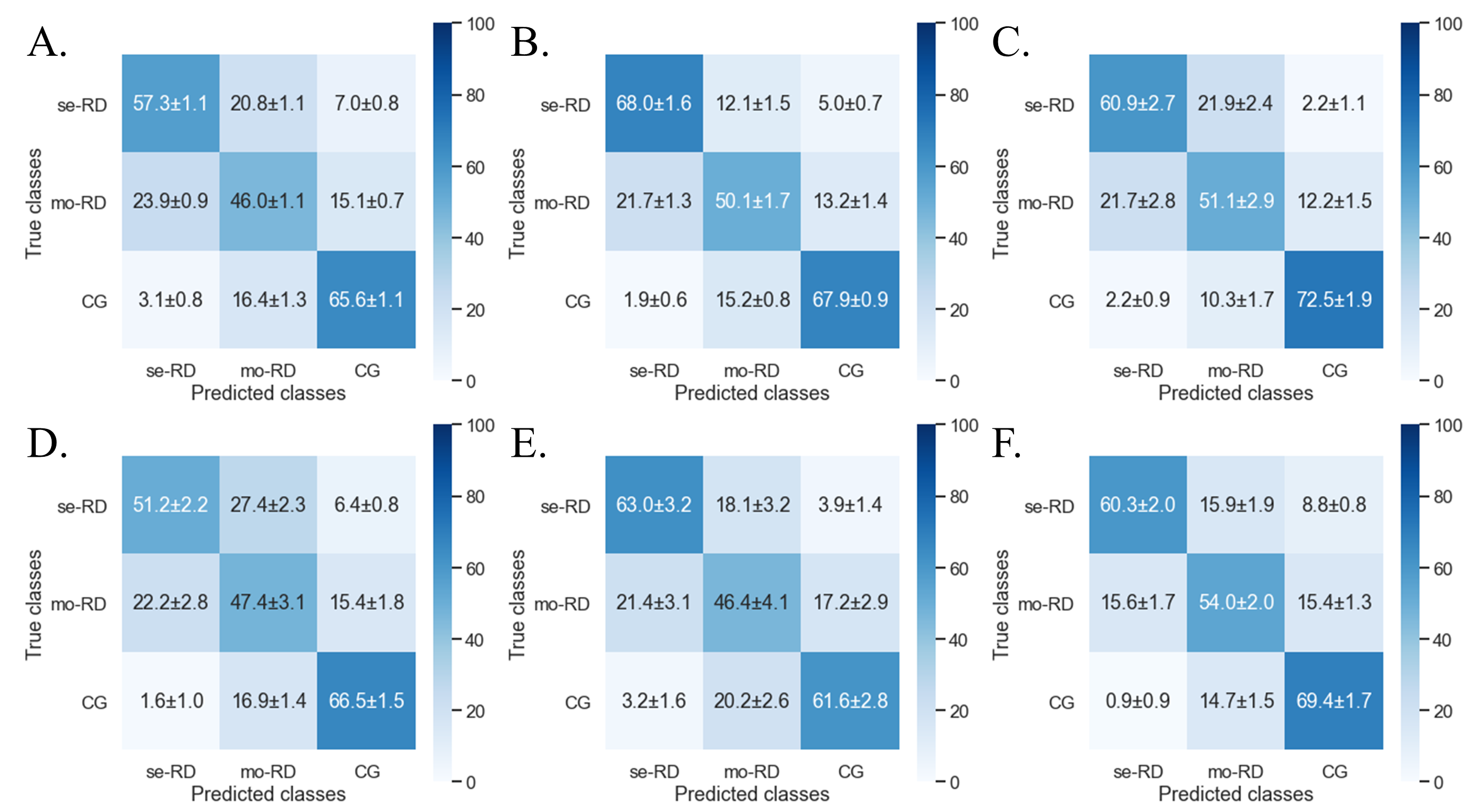


Supplementary Figure S7. Confusion matrices show the status of multiclass classifications using all imaging methods (ALL IMs) with linear discriminant analysis (LDA) (**A**); support vector machine (SVM) with linear (**B**), rbf (**C**) and sigmoid (**D**) kernels; decision tree (DT) (**E**); and random forest (RF) (**F**) classifiers, in classifying the three groups of chronic kidney disease. Severe renal dysfunction group (se-RD, eGFR < 30 mL/min/1.73 m²), moderate renal dysfunction group (mo-RD, eGFR ≥ 30 and < 60 mL/min/1.73 m^2^), and control group (CG, eGFR ≥ 60 mL/min/1.73 m^2^). The data are expressed as means ± standard deviations.


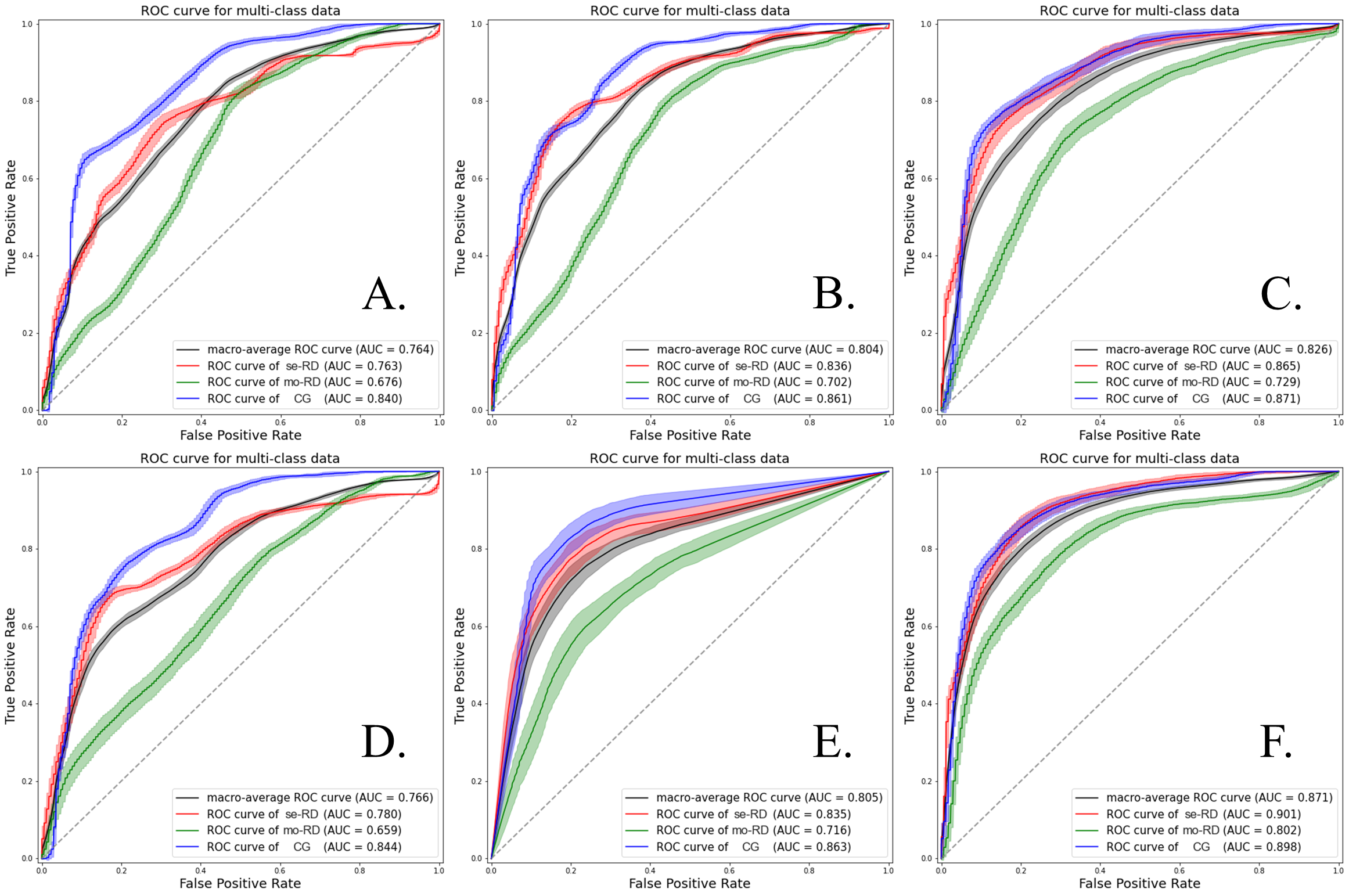


Supplementary Figure S8. The ROC curves and AUC values of multiclass classifications using T1-weighted in-phase (IP) image with linear discriminant analysis (LDA) (**A**); support vector machine (SVM) with linear (**B**), rbf (**C**) and sigmoid (**D**) kernels; decision tree (DT) (**E**); and random forest (RF) (**F**) classifiers, in classifying the three groups of chronic kidney disease. Severe renal dysfunction group (se-RD, eGFR < 30 mL/min/1.73 m²), moderate renal dysfunction group (mo-RD, eGFR ≥ 30 and < 60 mL/min/1.73 m^2^), and control group (CG, eGFR ≥ 60 mL/min/1.73 m^2^).


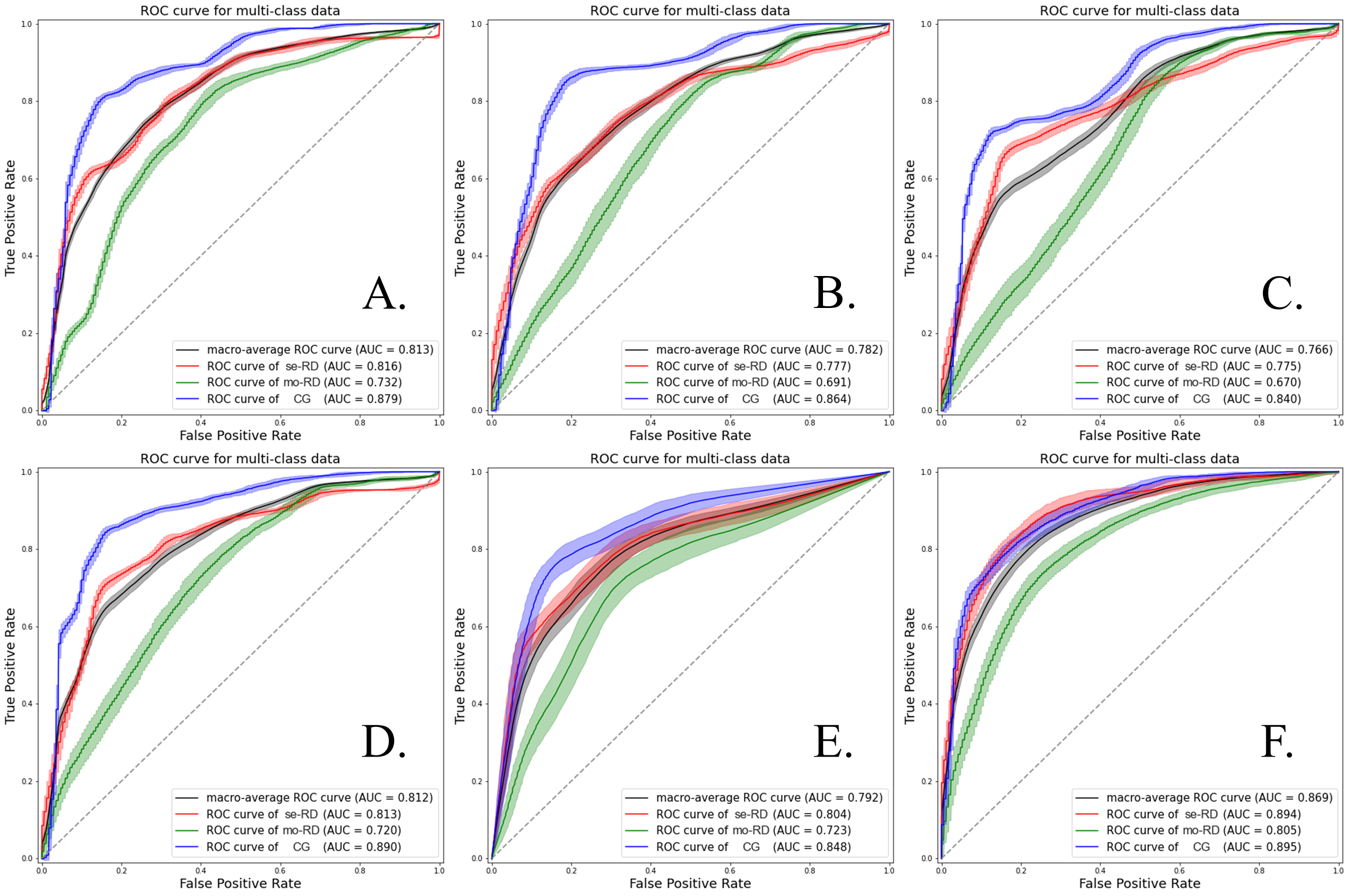


Supplementary Figure S9. The ROC curves and AUC values of multiclass classifications using T1-weighted opposed-phase (OP) image with linear discriminant analysis (LDA) (**A**); support vector machine (SVM) with linear (**B**), rbf (**C**) and sigmoid (**D**) kernels; decision tree (DT) (**E**); and random forest (RF) (**F**) classifiers, in classifying the three groups of chronic kidney disease. Severe renal dysfunction group (se-RD, eGFR < 30 mL/min/1.73 m²), moderate renal dysfunction group (mo-RD, eGFR ≥ 30 and < 60 mL/min/1.73 m^2^), and control group (CG, eGFR ≥ 60 mL/min/1.73 m^2^).


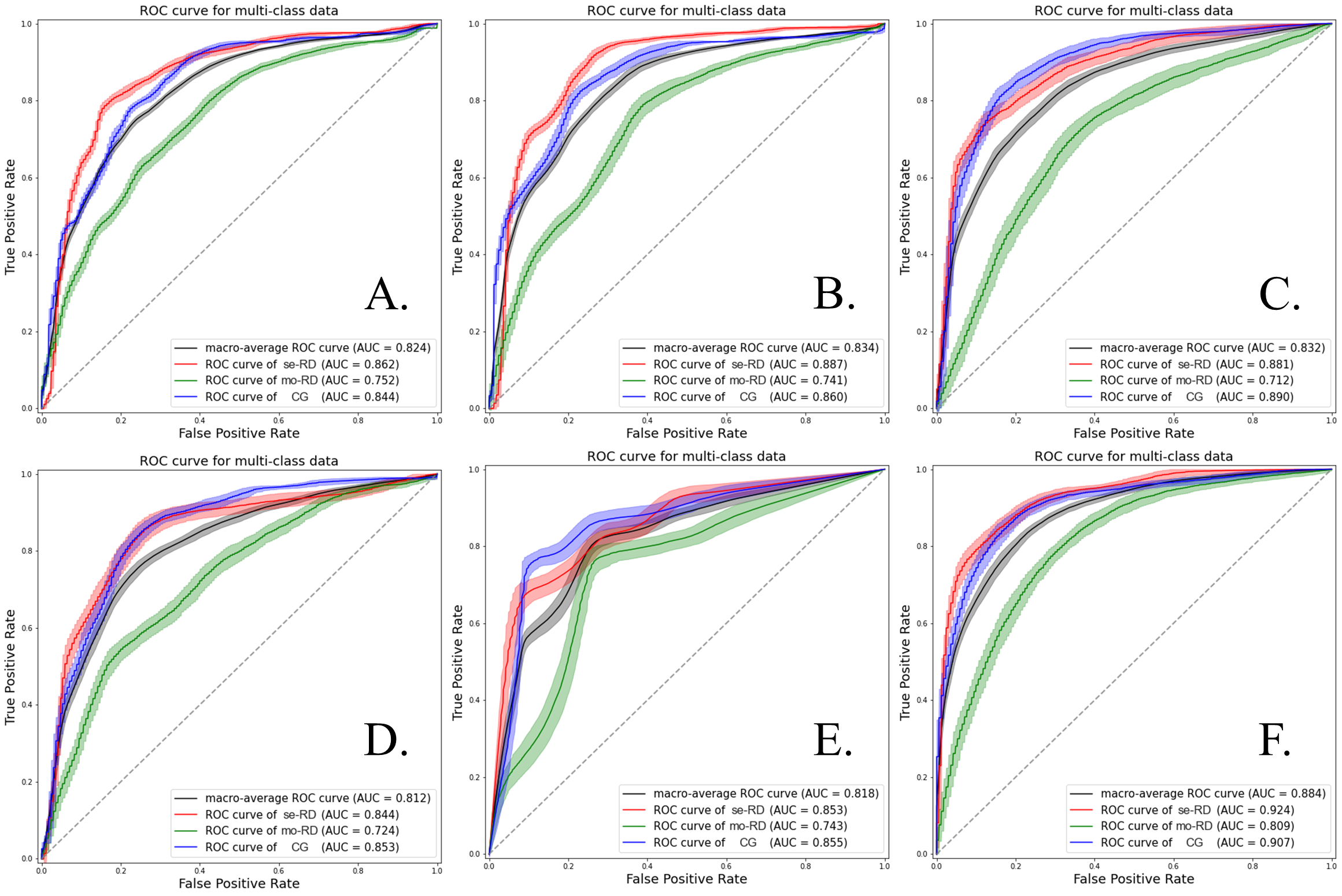


Supplementary Figure S10. The ROC curves and AUC values of multiclass classifications using T1-weighted water-only (WO) image with linear discriminant analysis (LDA) (**A**); support vector machine (SVM) with linear (**B**), rbf (**C**) and sigmoid (**D**) kernels; decision tree (DT) (**E**); and random forest (RF) (**F**) classifiers, in classifying the three groups of chronic kidney disease. Severe renal dysfunction group (se-RD, eGFR < 30 mL/min/1.73 m²), moderate renal dysfunction group (mo-RD, eGFR ≥ 30 and < 60 mL/min/1.73 m^2^), and control group (CG, eGFR ≥ 60 mL/min/1.73 m^2^).


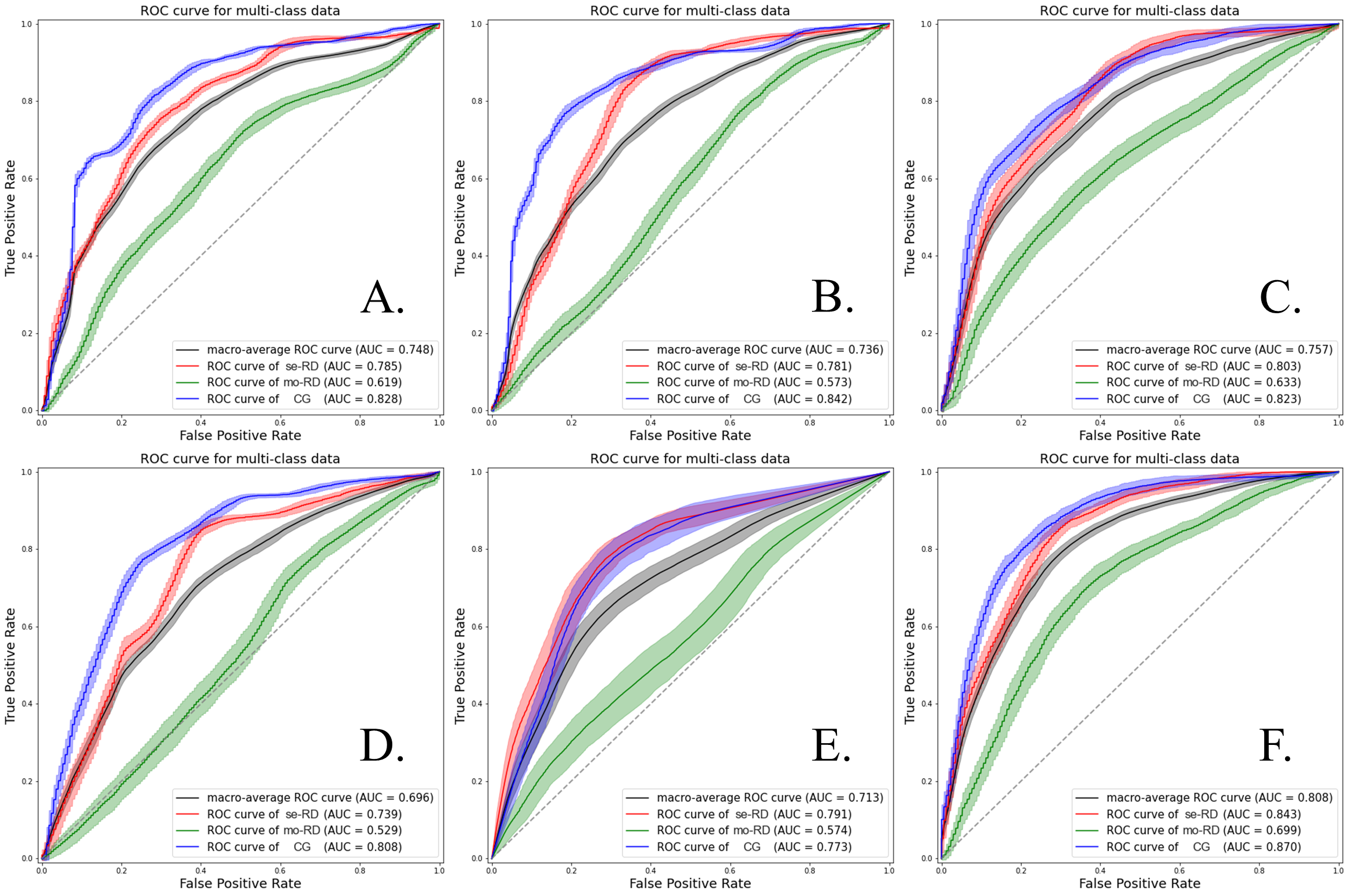


Supplementary Figure S11. The ROC curves and AUC values of multiclass classifications using apparent diffusion coefficient (ADC) map with linear discriminant analysis (LDA) (**A**); support vector machine (SVM) with linear (**B**), rbf (**C**) and sigmoid (**D**) kernels; decision tree (DT) (**E**); and random forest (RF) (**F**) classifiers, in classifying the three groups of chronic kidney disease. Severe renal dysfunction group (se-RD, eGFR < 30 mL/min/1.73 m²), moderate renal dysfunction group (mo-RD, eGFR ≥ 30 and < 60 mL/min/1.73 m^2^), and control group (CG, eGFR ≥ 60 mL/min/1.73 m^2^).


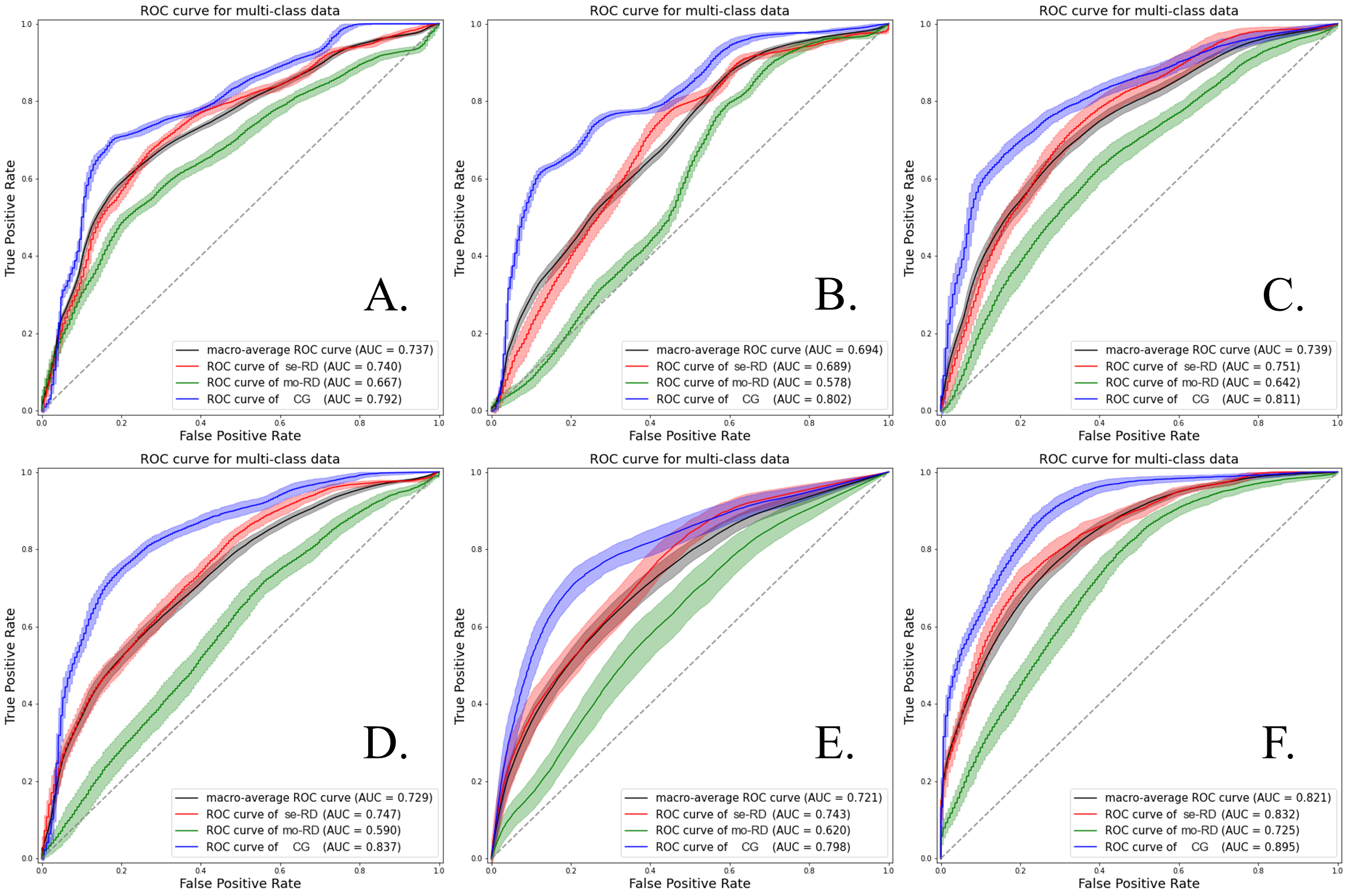


Supplementary Figure S12. The ROC curves and AUC values of multiclass classifications using T2* map with linear discriminant analysis (LDA) (**A**); support vector machine (SVM) with linear (**B**), rbf (**C**) and sigmoid (**D**) kernels; decision tree (DT) (**E**); and random forest (RF) (**F**) classifiers, in classifying the three groups of chronic kidney disease. Severe renal dysfunction group (se-RD, eGFR < 30 mL/min/1.73 m²), moderate renal dysfunction group (mo-RD, eGFR ≥ 30 and < 60 mL/min/1.73 m^2^), and control group (CG, eGFR ≥ 60 mL/min/1.73 m^2^).


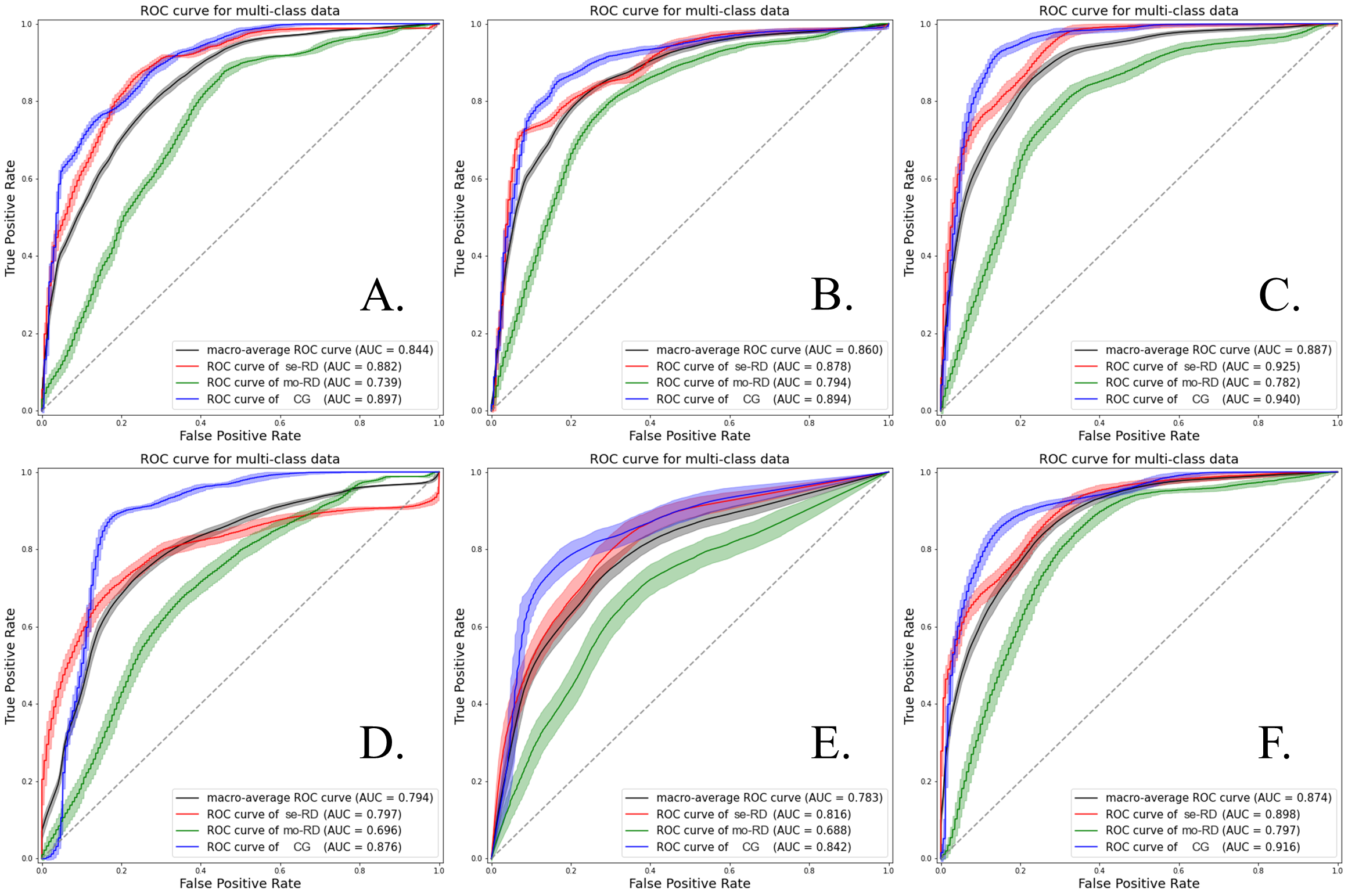


Supplementary Figure S13. The ROC curves and AUC values of multiclass classifications using all T1-weighted images (ALL T1WIs) with linear discriminant analysis (LDA) (**A**); support vector machine (SVM) with linear (**B**), rbf (**C**) and sigmoid (**D**) kernels; decision tree (DT) (**E**); and random forest (RF) (**F**) classifiers, in classifying the three groups of chronic kidney disease. Severe renal dysfunction group (se-RD, eGFR < 30 mL/min/1.73 m²), moderate renal dysfunction group (mo-RD, eGFR ≥ 30 and < 60 mL/min/1.73 m^2^), and control group (CG, eGFR ≥ 60 mL/min/1.73 m^2^).


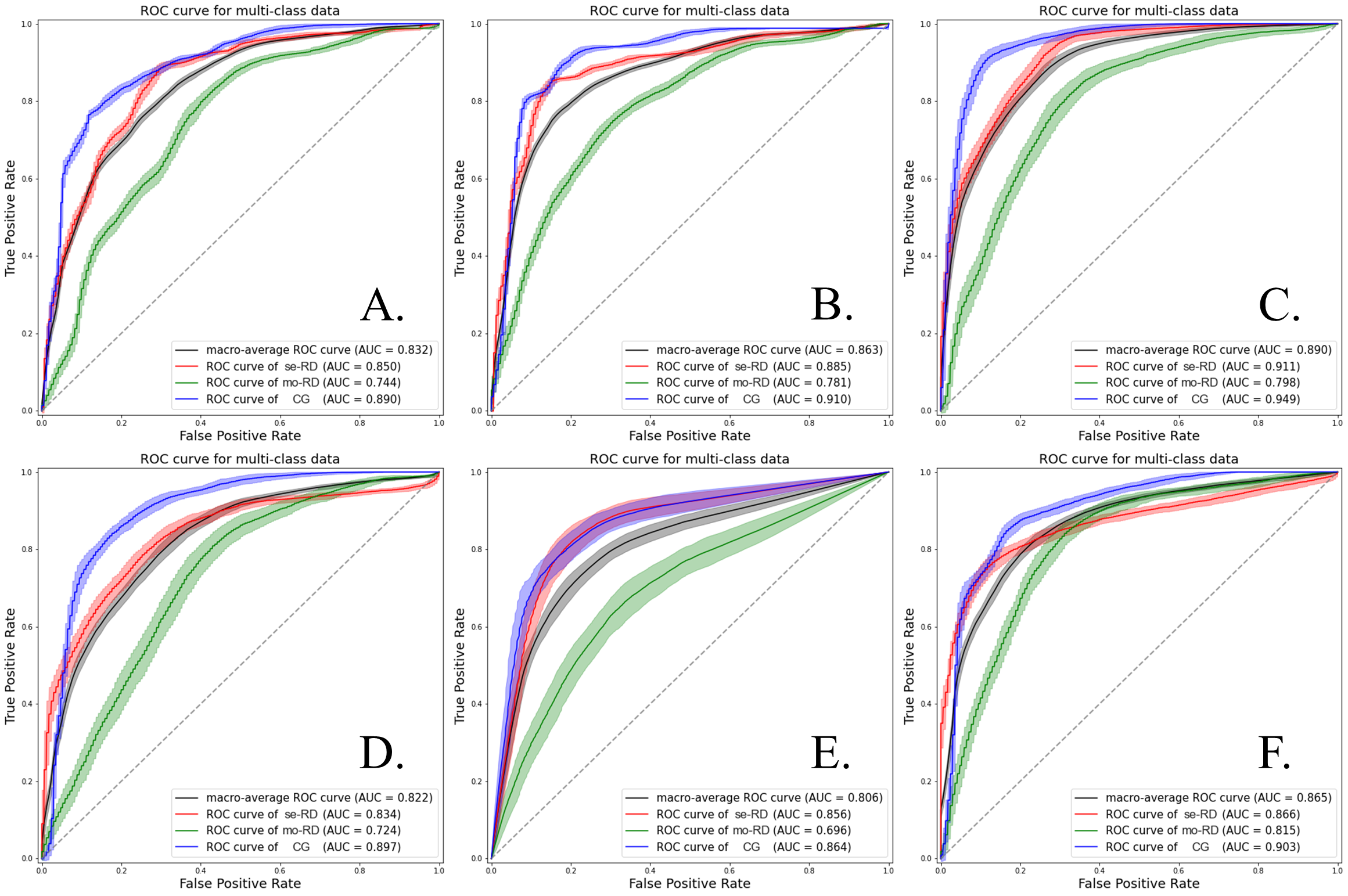


Supplementary Figure S14. The ROC curves and AUC values of multiclass classifications using all imaging methods (ALL IMs) with linear discriminant analysis (LDA) (**A**); support vector machine (SVM) with linear (**B**), rbf (**C**) and sigmoid (**D**) kernels; decision tree (DT) (**E**); and random forest (RF) (**F**) classifiers, in classifying the three groups of chronic kidney disease. Severe renal dysfunction group (se-RD, eGFR < 30 mL/min/1.73 m²), moderate renal dysfunction group (mo-RD, eGFR ≥ 30 and < 60 mL/min/1.73 m^2^), and control group (CG, eGFR ≥ 60 mL/min/1.73 m^2^).

The representative source codes for multiclass classification analysis:


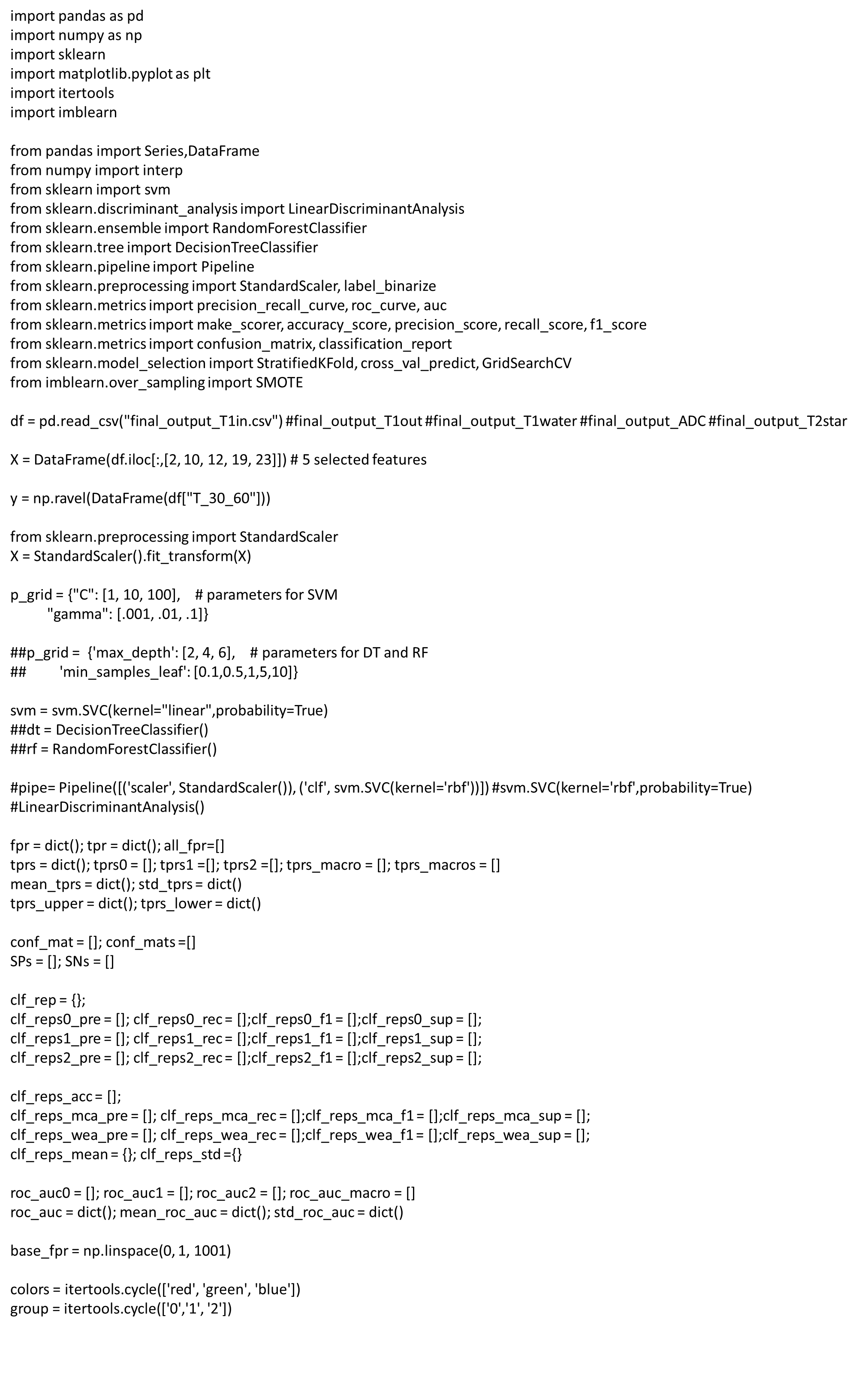


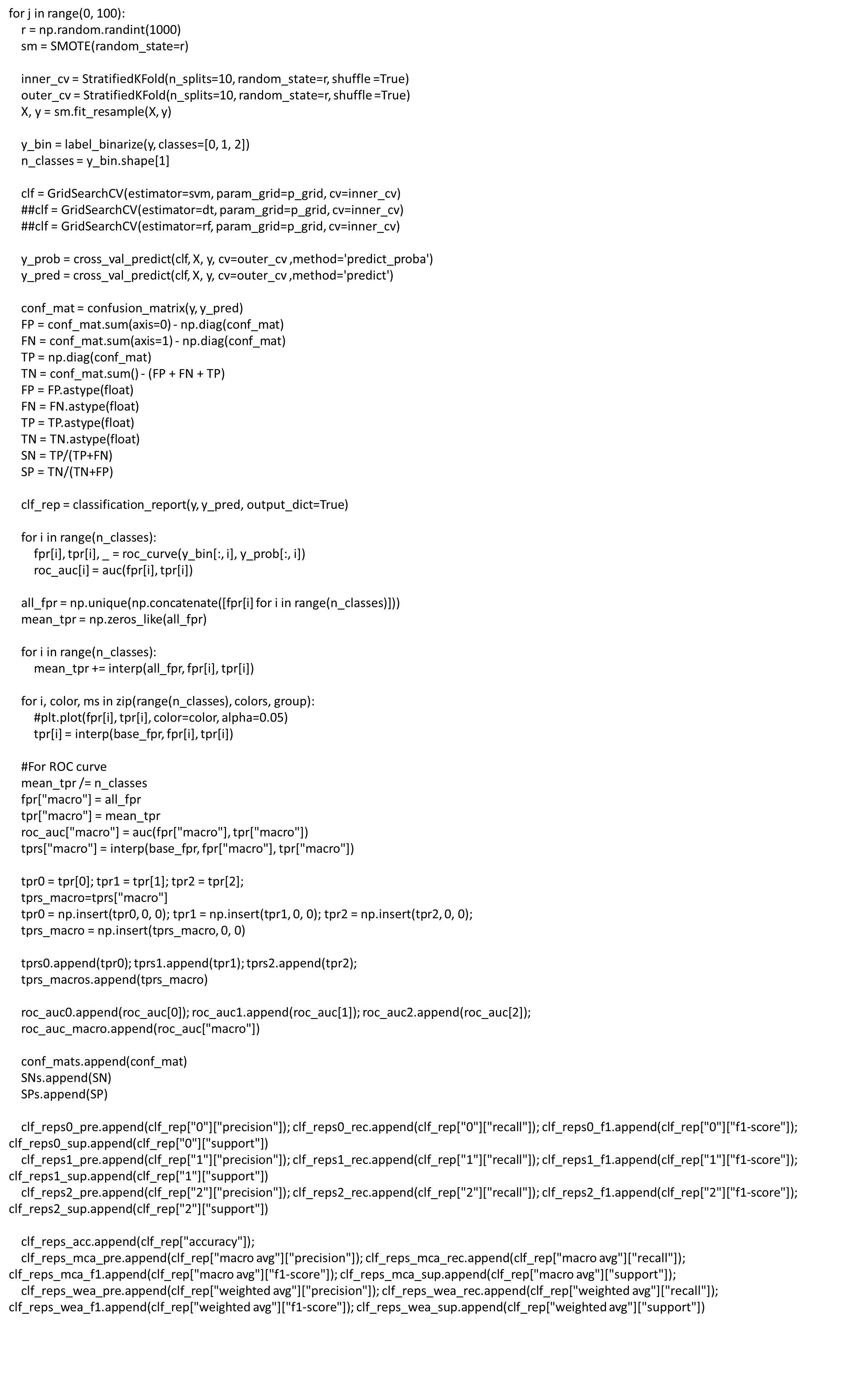


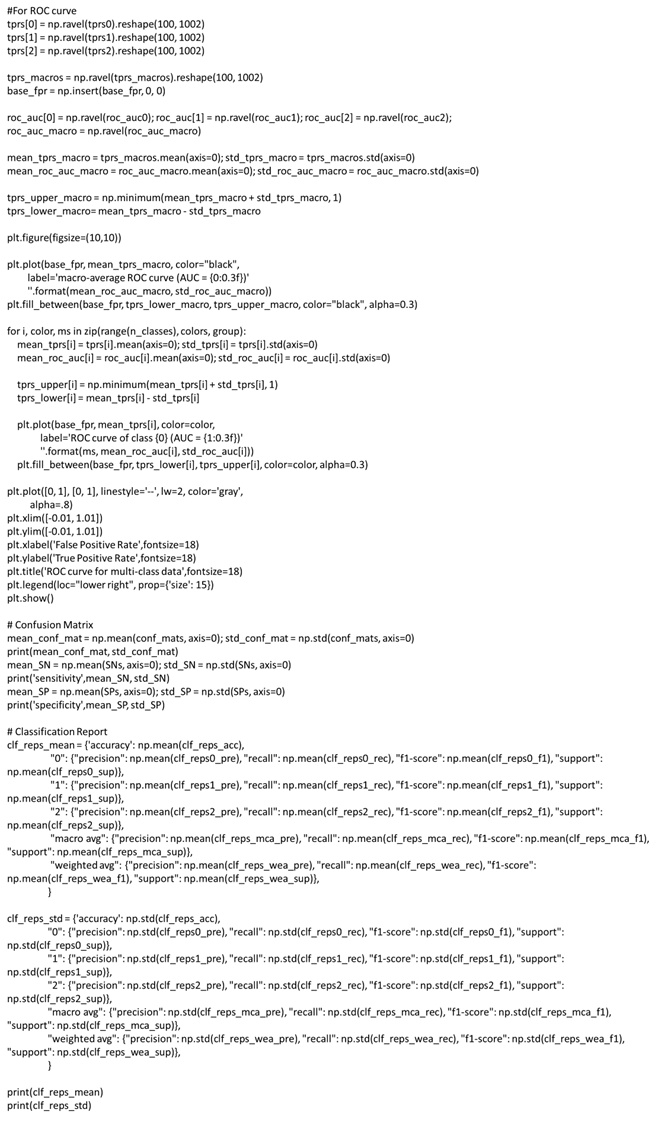


The anonymized data sets for multiclass classification analysis:

“final_output_T1in.csv”

“final_output_T1out.csv”

“final_output_T1water.csv”

“final_output_ADC.csv”

“final_output_T2star.csv”

(These CSV files were uploaded as Supplementary information online).
